# A Review on the Preparation Processes, Hot Deformation Behavior, and Microstructural Tailoring of the Ti-55531 Titanium Alloy

**DOI:** 10.3390/ma19214697

**Published:** 2026-11-04

**Authors:** Tianhui Wu, Yichao Gong, Zhijian Wei, Ming Liu, Miao Tian, Changsheng Tan, Ke Song, Zhanjun Yuan, Wen Guo, Guojun Zhang

**Affiliations:** 1 School of Materials Science & Engineering, Xi’an University of Technology, Xi’an 710048, Chinaliuming87@xaut.edu.cn (M.L.);; 2 AVIC Shaanxi Hongyuan Aviation Forging Co., Ltd., Xianyang 713801, China

**Keywords:** Ti-55531 titanium alloy, hot deformation behavior, microstructural evolution, dynamic recrystallization, microstructure tailoring

## Abstract

The Ti-55531 titanium alloy (Ti–5Al–5Mo–5V–3Cr–1Zr) is a typical near-β high-strength and high-toughness titanium alloy, featuring a combination of high strength, excellent hardenability, and a favorable strength–ductility balance. It holds significant application prospects in critical load-bearing structural components for aerospace vehicles and other high-end equipment. In recent years, extensive research has been conducted on the preparation processes, hot deformation behavior, and microstructure–property tailoring of this alloy. However, the relevant findings are widely dispersed in the literature, and a systematic understanding of the intrinsic interrelationships among preparation processes, thermomechanical behavior, and microstructural evolution is still lacking. This paper provides a comprehensive review of the research progress on the Ti-55531 titanium alloy. The development status of major preparation routes is summarized, including ingot metallurgy (with an emphasis on as-cast microstructural characteristics), powder metallurgy, and additive manufacturing. The hot deformation behavior and the effects of heat treatment processes on microstructural evolution are systematically reviewed, and research methodologies such as constitutive equations, processing maps, and numerical simulations are discussed. On this basis, the influence mechanisms of microstructural features (β grain size and α-phase morphology and distribution) on mechanical properties are analyzed, together with the microstructural evolution mechanisms during hot deformation, particularly dynamic recovery and dynamic recrystallization. Furthermore, key remaining challenges in current research, including microstructural evolution mechanisms, hot working instability behavior, and multi-scale simulation and prediction, are identified. Finally, future research directions are proposed in terms of process optimization, multi-scale modeling, and service performance investigation.

## 1. Introduction

With the rapid advancement of aerospace technology, next-generation aircraft and spacecraft impose increasingly stringent requirements on the overall mechanical and physical properties of structural materials. Particularly in the domains of large civil aircraft, space launch vehicles, and high-end equipment, critical load-bearing components are required not only to possess high strength and fracture toughness, but also to exhibit excellent fatigue resistance, corrosion resistance, and low density. Consequently, the development of advanced structural materials that synergistically combine high strength, high toughness, and low density has become a major research direction in the field of aerospace materials. Titanium alloys, owing to their high specific strength, outstanding corrosion resistance, and superior high-temperature performance, are extensively utilized in aircraft landing gear, airframe structures, and critical engine components [1,2,3], thereby occupying a pivotal position within the aerospace structural material system.

Among the numerous titanium alloy systems, near-β titanium alloys have garnered extensive attention due to their outstanding overall performance. In comparison with conventional α + β titanium alloys (e.g., Ti-6Al-4V), near-β titanium alloys generally contain higher contents of β-stabilizing elements (such as Mo, V, and Cr), endowing them with greater strength potential, deeper hardenability, and superior hot workability and forgeability. Among these, Ti-55531 titanium alloy is a typical near-β high-strength titanium alloy that, through optimized compositional design and appropriate heat treatment procedures [4], can yield a duplex microstructure consisting of primary α phase and fine secondary α precipitates, thereby achieving a favorable balance between strength and toughness. Relative to traditional titanium alloys, this alloy exhibits marked advantages in the fabrication of large-scale integral structural components, thus demonstrating promising application prospects for critical load-bearing aerospace parts [5,6,7,8].

However, current research on the Ti-55531 titanium alloy remains largely focused on individual processing routes or localized microstructural characterization, resulting in a lack of a systematic understanding of the intrinsic correlations among preparation processes, hot working behavior, and microstructural evolution. In particular, numerous issues concerning subtransus hot deformation microstructural mechanisms, multi-scale dynamic recrystallization behavior, microstructure control via additive manufacturing post-processing, and microstructural homogenization in complex components still require urgent and in-depth investigation. Furthermore, with the development of advanced manufacturing technologies and numerical simulation methods, achieving precise tailoring of the microstructure and mechanical properties of Ti-55531 titanium alloy has emerged as a critical research direction. In this context, systematically reviewing the research progress on preparation processes, hot working behavior, microstructural evolution mechanisms, and heat treatment optimization of Ti-55531 titanium alloy is of great significance for deepening the understanding of hot deformation and microstructural evolution in near-β titanium alloys, optimizing processing parameters, and promoting their engineering application in high-end aerospace equipment.

### 1.1. Compositional Design

In the Ti-55531 titanium alloy, the elemental composition ranges (in wt.%) are as follows: Al 4.0–6.0%, Mo 4.5–6.0%, V 4.5–6.0%, Cr 2.0–3.6%, Zr 0.3–2.0%, Fe 0.2–0.5%, with Ti as the balance [9]. Among these, Al serves as the primary α-stabilizing element [10], which enhances the strength of the α phase via solid-solution strengthening while broadening the processing window in the α + β two-phase region. The addition of Al facilitates the tailoring of the morphology and volume fraction of primary α (α_p_), thereby influencing the ductility and toughness of the alloy [11]. V, Mo, and Cr are the major β-stabilizing elements, and their synergistic effect endows the alloy with high β-phase stability. These elements exhibit considerable solubility in the β phase, significantly lowering the martensitic transformation temperature. This enables the alloy to retain a fully metastable β phase after quenching and simultaneously imparts excellent hardenability to Ti-55531 titanium alloy, which meets the through-thickness strengthening requirements for large forgings. The addition of Zr, which replaces Fe compared to the similar Ti5553 (Ti–5Al–5Mo–5V–3Cr–0.5Fe) alloy, is a key compositional feature. This substitution effectively avoids “β-spot” defects caused by Fe-rich segregation, thereby improving the metallurgical quality and the consistency of mechanical properties [12]. Moreover, Zr enhances the corrosion resistance and microstructural homogeneity of the alloy [13]. The content of Fe, a strong β-stabilizing element, is reduced relative to that in the BT22 (Ti-5Al-5Mo-5V-1Cr-1Fe) alloy to mitigate the tendency for compositional segregation. Furthermore, the contents of interstitial elements such as oxygen (O), nitrogen (N), and hydrogen (H) should be strictly controlled (O ≤ 0.20%, N ≤ 0.05%, H ≤ 0.015%) to suppress the formation of brittle phases and to improve ductility.

### 1.2. Phase Transformation Characteristics

The β-transus temperature (T_β_) of Ti-55531 titanium alloy is influenced by variations in its actual chemical composition, initial microstructure, and characterization methods, resulting in discrepancies among reported values. For instance, Warchomicka et al. [14] reported a T_β_ of approximately 803 °C for the Ti-55531 titanium alloy investigated, whereas Huang et al. [15] determined a value of approximately 830 ± 5 °C using differential thermal analysis in conjunction with microstructural observations. Xiang et al. [16] reported a T_β_ of 845 ± 5 °C, while values reported in other studies on Ti-55531 titanium alloy generally fall within the range of 835–847 °C [8,17]. Therefore, when comparing hot deformation behavior across different studies, greater emphasis should be placed on the deformation temperature relative to the respective T_β_ and the corresponding phase field, rather than solely on the absolute deformation temperature. At temperatures above T_β_, the microstructure consists predominantly of a single β phase; upon cooling into the α + β two-phase region, the β phase begins to transform and precipitate the α phase. By controlling the heat treatment temperature and cooling rate, various α-phase morphologies—such as primary α, secondary lamellar α, or fine dispersed α precipitates—can be obtained. The volume fraction, size, and spatial distribution of the α phase exert a significant influence on the mechanical properties of the alloy [18]. In general, a fine and uniformly distributed α phase markedly enhances the strength of the alloy, whereas an appropriate amount of primary α is beneficial for improving ductility and fracture toughness [19]. Consequently, through careful design of heat treatment schedules in the α + β phase field, the microstructure of Ti-55531 titanium alloy can be effectively tailored.

### 1.3. Main Application Areas

The Ti-55531 titanium alloy, characterized by high strength, excellent hardenability, and superior fatigue resistance [15,20], has found extensive applications in the aerospace sector. It is particularly suited for the manufacture of large load-bearing components that demand stringent strength requirements and substantial weight reduction. The alloy is also widely employed in various structural parts and critical connecting devices such as in landing gear, wings, and engine pylons [21,22]. In addition, Ti-55531 titanium alloy is used for important structural components such as rocket casings, meeting the combined requirements of strength, toughness, and durability under complex service conditions. With the advancement of manufacturing technologies for large integral structural components, the application prospects of Ti-55531 titanium alloy in high-load aerospace structures continue to expand [8,23].

### 1.4. Research and Characterization Methods

Investigations into the preparation processes, hot deformation behavior, and microstructure–property relationships of Ti-55531 titanium alloy generally require a combination of thermomechanical testing, microstructural characterization, mechanical testing, and numerical simulation. These complementary approaches provide experimental evidence at different scales, ranging from macroscopic deformation behavior to microstructural characteristics and mechanical properties, and play an important role in elucidating the effects of processing parameters on microstructural evolution and material performance.

For the investigation of hot deformation behavior, isothermal hot compression experiments are commonly conducted using thermomechanical simulators, such as the Gleeble system, over a range of deformation temperatures and strain rates. The resulting true stress–true strain curves are used to characterize the flow behavior and dynamic softening characteristics of the alloy. On this basis, Arrhenius-type constitutive models, the dynamic materials model (DMM), and processing maps are employed to establish relationships between deformation parameters and flow stress and to evaluate deformation behavior under different processing conditions. In addition, finite element simulations can be used to predict temperature, stress, and strain fields, as well as microstructural distributions during hot working, providing guidance for the optimization of practical forming processes.

Regarding microstructural characterization, optical microscopy (OM) and scanning electron microscopy (SEM) are widely employed to examine the microstructural features of Ti-55531 titanium alloy. OM is primarily used to characterize the morphology of prior β grains, general microstructural features, and the spatial distribution of deformation-induced microstructures. SEM enables more detailed characterization of the size, morphology, and distribution of the α phase and is also employed for fractographic observations and fracture behavior analysis. X-ray diffraction (XRD) is mainly used to identify the constituent phases and characterize changes in phase constitution following heat treatment. Electron backscatter diffraction (EBSD) provides crystallographic information on grain orientations, grain boundary distributions, and texture. Analyses based on inverse pole figure (IPF) maps, kernel average misorientation (KAM), grain orientation spread (GOS), and the distributions of low-angle grain boundaries (LAGBs) and high-angle grain boundaries (HAGBs) provide microstructural evidence for identifying dynamic recovery (DRV), continuous dynamic recrystallization (CDRX), and discontinuous dynamic recrystallization (DDRX). Transmission electron microscopy (TEM), offering higher spatial resolution, enables the observation of fine α precipitates, dislocations, and subgrain structures. It also facilitates detailed examination of α/β interfacial characteristics and their relationships with microstructural evolution and strengthening mechanisms.

For mechanical property evaluation, tensile, compression, and hardness tests are commonly performed to characterize the strength, ductility, and deformation response of Ti-55531 titanium alloy under different processing and heat treatment conditions. When combined with microstructural characterization, these measurements enable correlations to be established between mechanical properties and microstructural parameters, including β-grain size, α-phase morphology and distribution, and phase boundary characteristics.

Overall, research on Ti-55531 titanium alloy has progressively adopted an integrated methodology based on hot deformation experiments, supported by multiscale microstructural characterization and complemented by mechanical testing and numerical simulation. The information obtained from these techniques is mutually complementary. Particularly in the investigation of dynamic recrystallization mechanisms, flow stress curves or individual microstructural parameters alone are generally insufficient to reliably identify the predominant mechanism. A comprehensive assessment incorporating multiple sources of microstructural evidence, including grain morphology, grain boundary characteristics, and local crystallographic orientation variations, is therefore essential.

**Figure 1:**
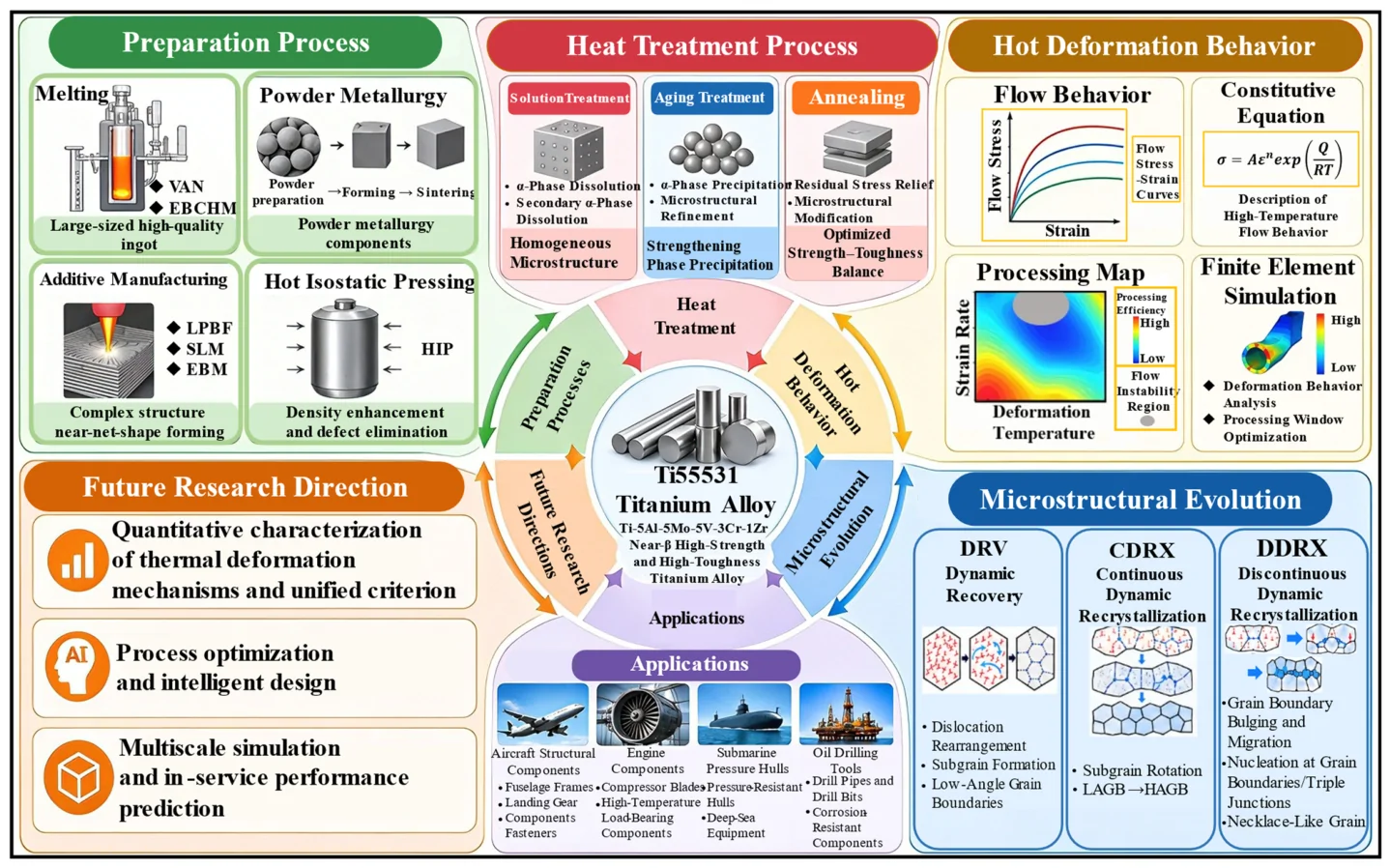
Schematic illustration of the overall framework of this review on Ti-55531 titanium alloy.

Generative AI tools were used to assist in the preparation of Figure 1, specifically for developing the overall graphical framework and generating several small schematic illustrations. The remaining elements were prepared, organized, and edited by the authors based on the content of the manuscript. All AI-assisted elements were reviewed and revised by the authors to ensure the scientific accuracy and consistency of the final figure.

### 1.5. Main Contents and Framework

This review focuses on the near-β high-strength Ti-55531 titanium alloy and systematically summarizes the research progress concerning its preparation processes, hot working behavior, and the relationships between microstructure and mechanical properties [24]. In recent years, substantial investigations have been conducted by researchers globally on the preparation technologies, heat treatment procedures, hot deformation response, and microstructural evolution of Ti-55531 titanium alloy, yielding significant findings. 

Accordingly, the main research contents and overall framework of this review are illustrated in Figure 1. In this context, the present review first introduces the developmental background of Ti-55531 titanium alloy and its typical applications in aerospace, marine engineering, and high-end equipment sectors. Subsequently, the major preparation routes, including ingot metallurgy/forging, powder metallurgy, and additive manufacturing, are reviewed, with an analysis of the effects of different processing techniques on the resulting microstructure and properties. On this basis, the hot deformation behavior of the alloy is summarized, with particular emphasis on forging methods, heat treatment regimes, and research methodologies such as constitutive equations, processing maps, and numerical simulations. Following that, the microstructural evolution mechanisms during hot deformation, specifically dynamic recovery and dynamic recrystallization, are systematically reviewed. Finally, building upon a synthesis of existing findings, future research directions are proposed from the perspectives of thermomechanical processing mechanisms, microstructural tailoring, and advanced manufacturing technologies, with the aim of providing a reference for the engineering application of Ti-55531 titanium alloy in high-end aerospace and related fields.

## 2. Preparation Technologies and Initial Microstructure of the Ti-55531 Titanium Alloy

### 2.1. Melting and As-Cast Microstructural Characteristics

**Figure 2:**
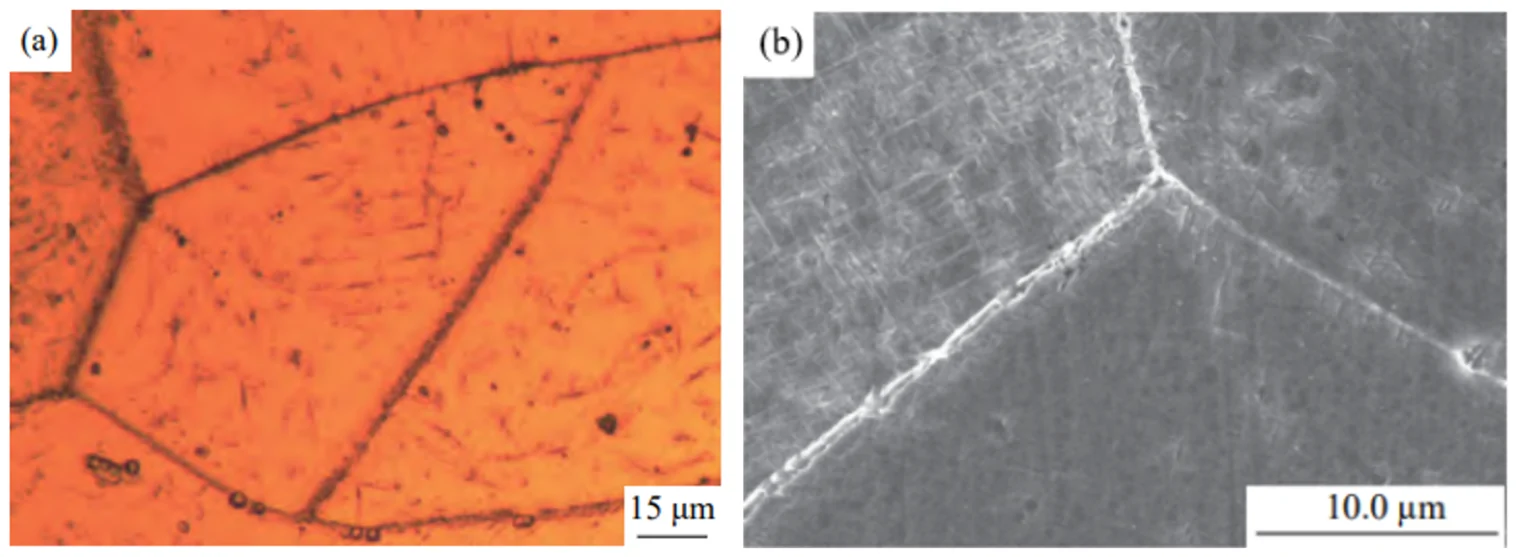
As-cast microstructure of Ti-55531 titanium alloy: (**a**) optical micrograph, (**b**) SEM image. Reproduced with permission from Ref. [27]. Copyright 2025, Youke Publishing.

The Ti-55531 titanium alloy is typically fabricated via vacuum arc remelting (VAR) or electron beam cold hearth melting (EBCHM) to ensure compositional homogeneity and to reduce gaseous and inclusion-type defects [25,26]. During the melting process, due to the relatively rapid solidification rate of the molten pool and the steep temperature gradients, the ingot microstructure frequently exhibits pronounced columnar grains or coarse equiaxed grains, accompanied by a certain degree of dendritic segregation. As a result, β-stabilizing elements (e.g., Mo, V, and Cr) tend to enrich in the interdendritic regions, whereas the α-stabilizing element Al exhibits a relatively uniform distribution. During solidification, the Ti-55531 titanium alloy first solidifies as a single β phase, followed by a β → α phase transformation upon cooling, resulting in a grain-boundary α phase and intragranular lamellar or acicular α precipitates. As shown in the optical micrograph in Figure 2a, the as-cast microstructure is primarily composed of relatively large β grains with well-defined polygonal grain boundaries, and distinct second-phase distributions are observable at local grain boundaries, indicating that a certain fraction of grain-boundary α has already formed during solidification and subsequent cooling. Concurrently, finer microstructural features or transformation products are visible within the grain interiors, reflecting the presence of compositional segregation and transformation structures in the as-cast state. The SEM image in Figure 2b provides a clearer view of the grain-boundary region: the boundaries exhibit a continuous or semi-continuous bright contrast, particularly pronounced at grain-boundary triple junctions, which is closely associated with the precipitation of grain-boundary α; in contrast, the grain interiors appear relatively featureless, containing only a few fine precipitates or slip traces, indicating that the overall as-cast microstructure remains predominantly β-matrix based [27]. The above observations demonstrate that the Ti-55531 titanium alloy in the as-cast condition is characterized by the typical coexistence of coarse β grains and a grain-boundary α phase. However, the conventionally melted and solidified as-cast structure of Ti-55531 titanium alloy commonly suffers from issues such as coarse β grains, dendritic segregation, and continuous grain-boundary α distributions, and such microstructural inhomogeneity can exert a notable influence on subsequent hot working processes and mechanical properties. Therefore, in practical engineering applications, thermomechanical processing routes such as forging and rolling are generally required to break down and refine the as-cast structure, thereby obtaining a uniform and fine microstructure [28]. With the development of advanced materials fabrication technologies, and in an effort to overcome the limitations of conventional ingot metallurgy—including microstructural segregation, prolonged processing cycles, and difficulties in forming complex geometries—researchers have begun to explore alternative processing routes. Among these, powder metallurgy, which enables precise compositional control and near-net-shape forming [29], has attracted growing attention for the microstructure tailoring and property optimization of Ti-55531 titanium alloy, and has emerged as a significant processing pathway beyond conventional ingot metallurgy. 

### 2.2. Powder Metallurgy Approach

Powder metallurgy (PM) involves the fabrication of dense alloy materials or components via the preparation of metal or alloy powders, followed by compaction and high-temperature sintering. This technology offers an effective alternative route for titanium alloy production, significantly alleviating issues inherent in conventional forging, such as unstable forming quality, complex processing routes, and insufficient manufacturing flexibility [30]. Despite its advantage in compositional homogeneity, the PM route incurs relatively high production costs and is constrained by powder preparation and densification equipment. Consequently, its current application to large-scale aerospace load-bearing components remains limited, and it is primarily employed for fundamental investigations into microstructure control mechanisms and process optimization of Ti-55531 titanium alloy [31].

**Figure 3:**
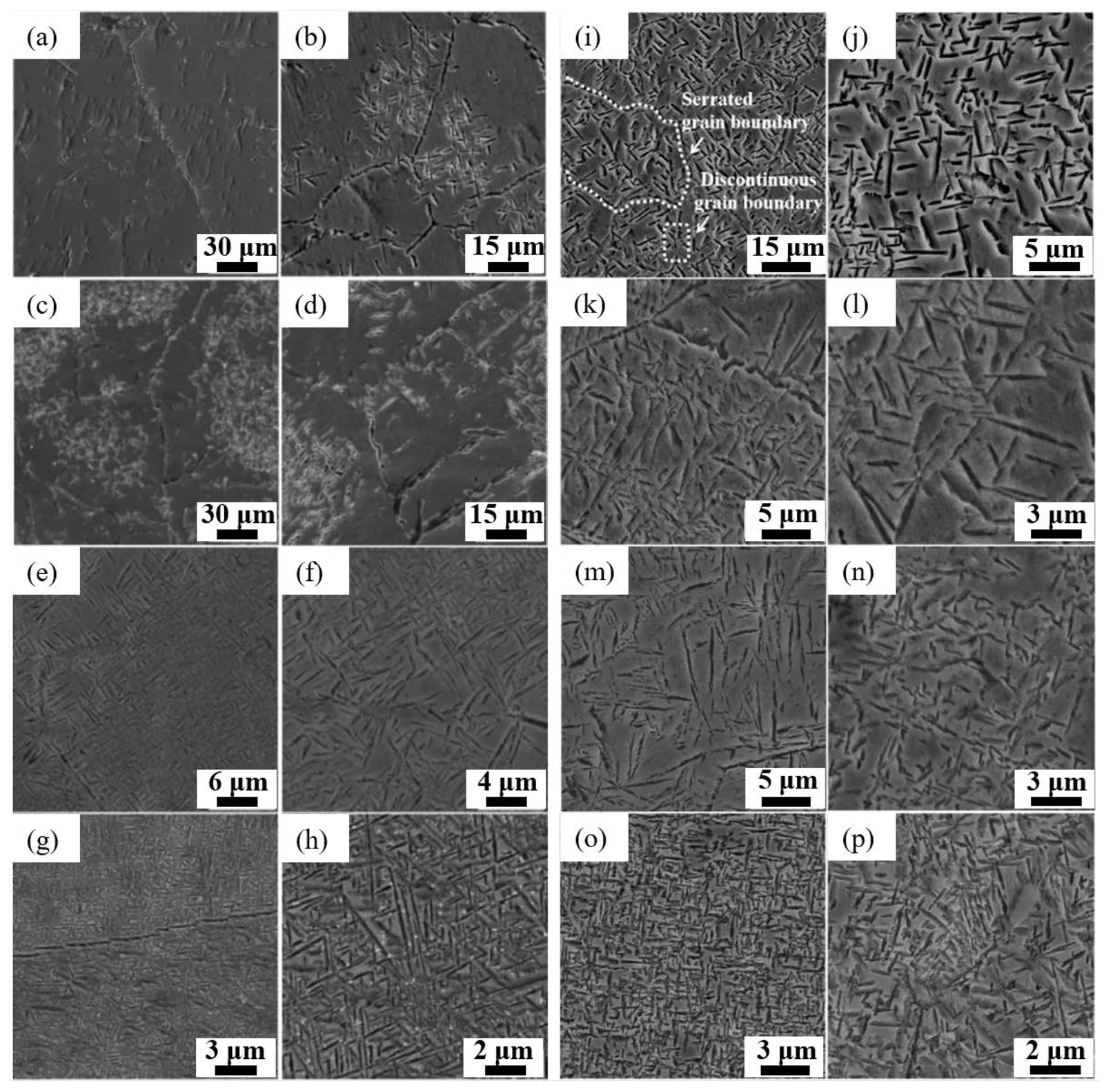
SEM micrographs of Ti-55531 titanium alloys fabricated by spark plasma sintering (SPS) using different powder conditions and in situ aging temperatures: (**a**–**h**) as-received powders; (**i**–**p**) ball-milled powders; (**a**,**b**,**i**,**j**) 750 °C; (**c**,**d**,**k**,**l**) 700 °C; (**e**,**f**,**m**,**n**) 650 °C; and (**g**,**h**,**o**,**p**) 600 °C. Reproduced from Ref. [32] with permission of the author.

Liu [32] fabricated the Ti-55531 titanium alloy by high-energy ball milling combined with spark plasma sintering (SPS) and systematically investigated the effects of ball milling and in-situ aging on the resulting microstructure and mechanical properties. Figure 3 presents a comparative microstructural analysis of SPS samples prepared from powders in different states. High-energy ball milling introduced a high density of dislocations within the powder particles, providing abundant nucleation sites for α-phase precipitation, which significantly accelerated the precipitation kinetics and promoted a more uniform spatial distribution of α precipitates. Compared with samples sintered from unmilled powders, those from milled powders exhibited substantially reduced or even eliminated precipitate-free zones (PFZs) in the vicinity of grain boundaries, indicative of improved microstructural homogeneity. Furthermore, by adjusting the in-situ aging temperature, the size and volume fraction of the α phase could be tailored, thereby enabling tunable combinations of strength and ductility. This study established a novel processing pathway for the PM fabrication of high-performance Ti-55531 titanium alloy.

**Figure 4:**
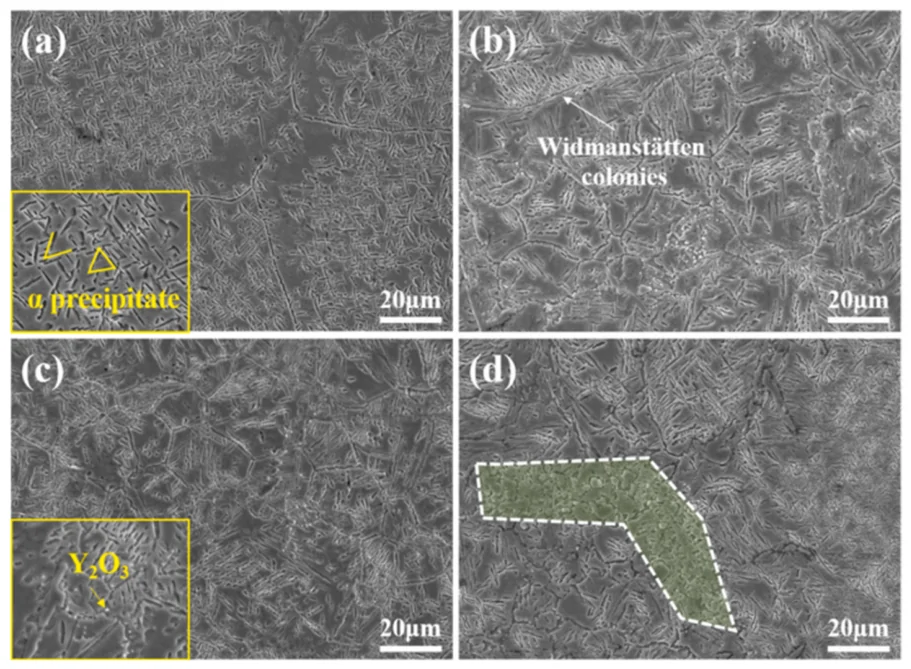
SEM micrographs of Ti-55531 titanium alloy with different Y contents after holding at 750 °C for 60 min: (**a**) Ti-55531; (**b**) Ti-55531-0.2Y; (**c**) Ti-55531-0.5Y; and (**d**) Ti-55531-1Y. The yellow boxes show enlarged views of α precipitates in (**a**) and Y_2_O_3_ particles in (**c**), while the green-shaded region in (**d**) highlights fine β grains without α precipitation. Reproduced from Ref. [33] with permission of the author.

Building upon PM techniques, Chen [33] introduced a rare-earth element Y into Ti-55531 titanium alloy during SPS processing and proposed a novel strategy for constructing a network microstructure. Their findings revealed that Y reacts with oxygen during sintering to form stable Y_2_O_3_ particles, which exert a pinning effect at grain boundaries, thereby suppressing β grain growth. As a result, coarse β grains become surrounded by fine β grains, forming a characteristic network structure (representative microstructures are shown in Figure 4). In contrast to a uniform microstructure, this network structure induces a gradient distribution of stress and strain during deformation: the fine-grained regions bear higher stresses, while the coarse-grained regions accommodate plastic strain, enabling a simultaneous improvement in both strength and ductility. This work demonstrates that the construction of heterogeneous structures through the combination of microalloying and PM processing is an effective strategy for overcoming the conventional strength–ductility trade-off, providing a new perspective for the microstructural design and performance optimization of Ti-55531 titanium alloy.

In recent years, increasing attention has also been directed towards integrated processing routes combining powder metallurgy with subsequent thermomechanical processing. Zhang et al. [29] fabricated Ti-55531 titanium alloy through powder metallurgy followed by hot rolling in the single-phase β region and subsequent aging treatment. By refining the β grains through hot deformation and controlling the precipitation of multiscale α phases, this processing route improved microstructural homogeneity while simultaneously enhancing strength and ductility.

Overall, powder metallurgy research on Ti-55531 titanium alloy has progressively evolved from conventional sintering-based densification towards microalloying, heterogeneous microstructure design, and the synergistic integration of powder metallurgy, thermomechanical processing, and heat treatment. Existing studies have demonstrated that these approaches can effectively control the size and distribution of β grains and α phases, thereby improving the strength–ductility balance. Nevertheless, further investigations are required to address the challenges associated with the fabrication of large-scale components, control of microstructural homogeneity, and industrial-scale implementation.

### 2.3. Additive Manufacturing

Additive manufacturing (AM), which relies on the layer-by-layer melting of metal powders using high-energy beams, has been progressively applied to the fabrication of the Ti-55531 titanium alloy, offering advantages such as short development cycles, tool-less production, and the ability to integrally form complex geometries [34]. Current AM research on this alloy primarily focuses on three technological routes: laser powder bed fusion (LPBF), selective laser melting (SLM), and electron beam melting (EBM). LPBF and SLM share similar working principles, in which a high-power laser beam selectively melts pre-laid metal powders layer by layer, enabling the direct fabrication of metallic components with complex geometries, high dimensional precision, and good surface quality, as well as lattice structures with regular porosity [35,36,37]. This approach represents the most widely adopted AM technology for Ti-55531 titanium alloy to date. EBM, in contrast, utilizes a high-energy electron beam as the heat source within a high-vacuum chamber to melt metal powders layer by layer, directly producing fully dense parts. The vacuum environment lowers manufacturing costs and improves efficiency for titanium components, while also offering the advantages of forming complex geometries, high material utilization, and rapid processing speeds, thereby enabling near-net-shape fabrication [38,39].

**Figure 5:**
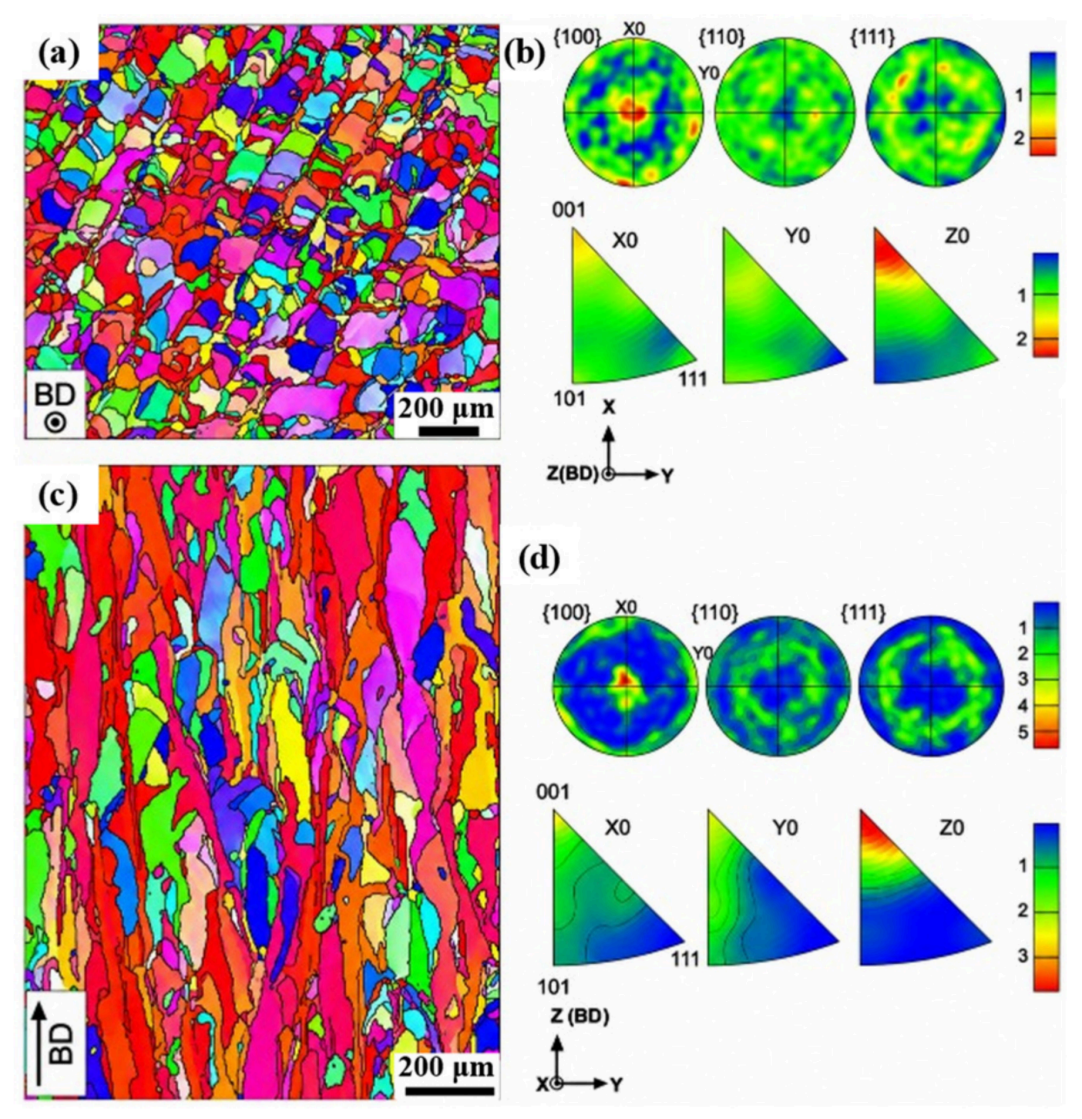
EBSD results of SLM-fabricated Ti-55531 titanium alloy along different orientations: (**a**,**b**) horizontal direction; (**c**,**d**) build direction; (**a**,**c**) IPF-Z maps; and (**b**,**d**) the corresponding pole figures and inverse pole figures. Reproduced from Ref. [4] with permission of the author.

**Figure 6:**
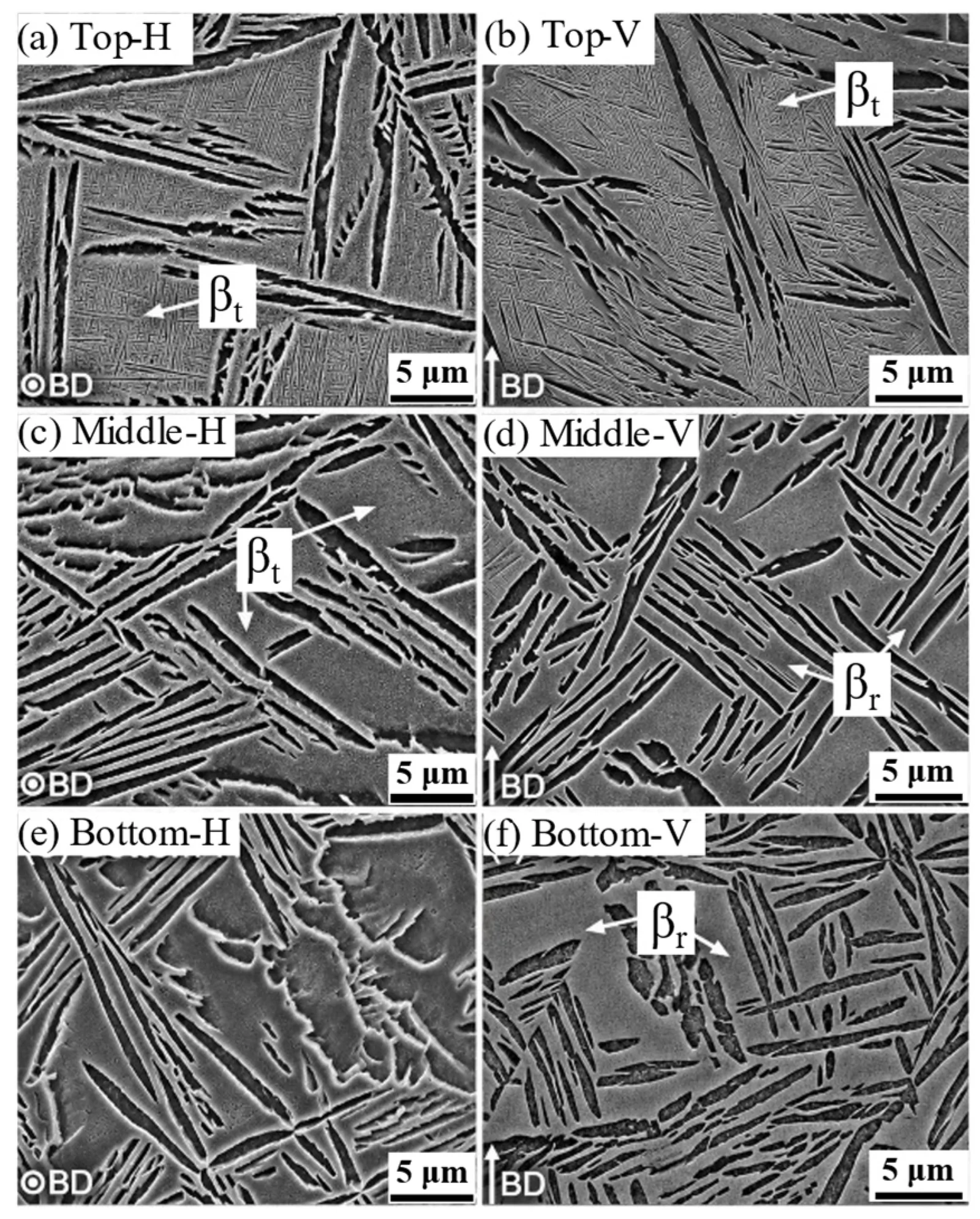
SEM secondary electron images of EBM-fabricated Ti-55531 titanium alloy at different build heights: (**a**,**b**) top region; (**c**,**d**) middle region; (**e**,**f**) bottom region. Reproduced from Ref. [4] with permission of the author.

Deng [4] fabricated the Ti-55531 titanium alloy via both SLM and EBM, and systematically investigated the microstructural evolution and mechanical properties. Their results indicate that under the rapid solidification conditions of SLM, the alloy develops columnar β grains that grow epitaxially along the build direction, accompanied by a pronounced <100>β texture (Figure 5). In contrast, during EBM processing, the relatively high preheating temperature and thermal accumulation result in a typical α + β lamellar structure, which exhibits a graded microstructural feature along the build direction (Figure 6), resulting in variations in strength and ductility with deposition height [40,41]. This study elucidated the intrinsic relationships among processing parameters, microstructural evolution, and mechanical properties in powder bed fusion (PBF) processes, providing valuable guidance for AM process optimization.

Compared with conventionally forged materials, Ti-55531 titanium alloy fabricated by AM exhibit distinct grain morphologies and texture evolution characteristics. During SLM, the high-energy laser beam induces rapid localized melting and solidification, generating pronounced directional thermal gradients within the melt pool. Consequently, β grains tend to grow epitaxially along the predominant heat-flow direction, resulting in columnar β grains elongated along the build direction and a strong <100>β preferred orientation [4]. Thus, the as-built texture is primarily governed by the heat-flow direction, epitaxial grain growth, and successive thermal cycling during solidification. In contrast, the texture of conventionally forged materials develops predominantly during high-temperature plastic deformation, with its evolution closely associated with slip system activation, lattice rotation, deformation band formation, dynamic recovery, and dynamic recrystallization. Variations in deformation temperature, strain rate, and strain can induce the redistribution of existing deformation-induced orientations, while recrystallization may further weaken or modify the deformation texture. Therefore, the texture differences between additively manufactured and conventionally forged Ti-55531 titanium alloy primarily originate from their distinct texture formation mechanisms: preferential grain growth during solidification dominates in AM materials, whereas plastic deformation and recrystallization govern texture evolution in forged materials. Subsequent heat treatment, hot isostatic pressing, or forging of AM components can further modify the columnar β-grain morphology and the initially strong texture, thereby reducing microstructural anisotropy in the as-built material.

**Figure 7:**
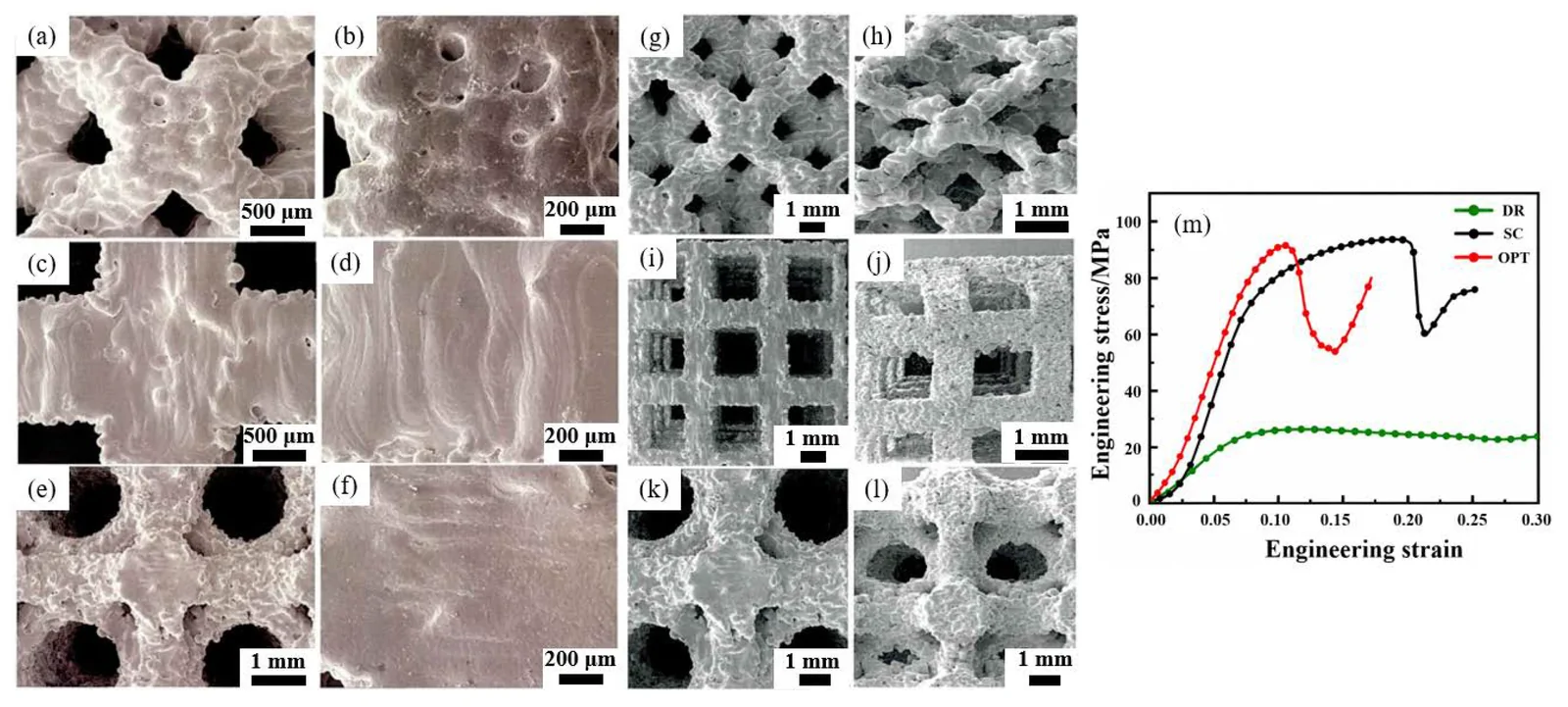
Surface morphologies and compressive properties of SLM-fabricated Ti-55531 titanium alloy with different lattice structures: (**a**,**b**) RD; (**c**,**d**) SC; (**e**,**f**) OPT; (**g**,**h**) RD, (**i**,**j**) SC, and (**k**,**l**) OPT structures before and after compression; and (**m**) compressive stress–strain curves. Reproduced from Ref. [42] with permission of the author.

In addition to process parameter optimization, structural design constitutes another important avenue for enhancing the mechanical performance of additively manufactured Ti-55531 titanium alloy. Zhao [42] employed SLM to fabricate Ti-55531 titanium alloy and systematically studied the influence of different lattice architectures on the microstructure and mechanical properties. Their findings reveal that distinct lattice configurations exhibit different molten-pool solidification behaviors and heat transfer characteristics during SLM processing, leading to significant differences in porosity, surface morphology, and mechanical performance. Among these, the optimized topology (OPT) lattice structure exhibits a more uniform pore distribution and lower defect content (representative surface morphologies shown in Figure 7), along with superior compressive strength and elastic modulus, achieving a compressive strength of 161 MPa—substantially higher than those of the rhombic dodecahedron (RD) and simple cubic (SC) structures. This work demonstrates that judicious lattice design can effectively enhance the mechanical properties of SLM-fabricated Ti-55531 titanium alloy, offering new insights into the design of lightweight additively manufactured titanium alloy structures.

**Figure 8:**
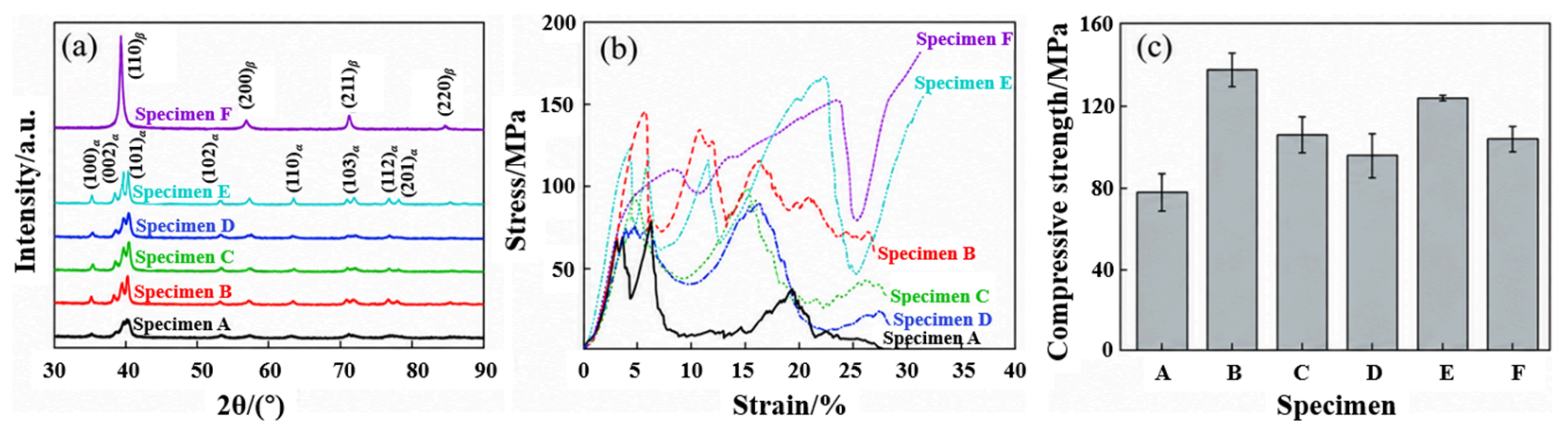
(**a**) XRD patterns of Ti-55531 titanium alloy porous material struts in the as-built condition and after different heat treatment regimens; (**b**,**c**) compressive stress–strain curves and compressive strength of heat-treated Ti-55531 titanium alloy porous materials. Specimen F denotes the as-built (non-heat-treated) specimen; all other specimens were subjected to designated heat treatment regimens. Reproduced with permission from Ref. [43]. Copyright 2023, Acta Metallurgica Sinica.

Building upon structural design optimization, heat treatment has emerged as another crucial approach for improving the performance of additively manufactured porous Ti-55531 titanium alloy structures. Zhang et al. [43] and Xu et al. [44] fabricated Ti-55531 titanium alloy via SLM and investigated the effects of heat treatment on the microstructure and mechanical properties. Their results demonstrate that the as-SLM microstructure is predominantly composed of the β phase, which transforms into an α + β duplex structure upon heat treatment. As shown in Figure 8, the as-built specimens exhibit ductile deformation behavior, whereas the heat-treated specimens display brittle metallic deformation characteristics, with heat treatment leading to enhanced strength. This study confirms that heat treatment is an effective route for optimizing the performance of additively manufactured Ti-55531 titanium alloy porous structures. Ye et al. [23] adopted a hybrid manufacturing approach combining LPBF with die forging to produce Ti-55531 titanium alloy, and the as-fabricated microstructure remained predominantly β phase. Collectively, the above studies—spanning process optimization, structural design, heat treatment regulation, and hybrid manufacturing—provide a substantial body of evidence for the microstructure–property tailoring of additively manufactured Ti-55531 titanium alloy.

### 2.4. Hot Isostatic Pressing

**Figure 9:**
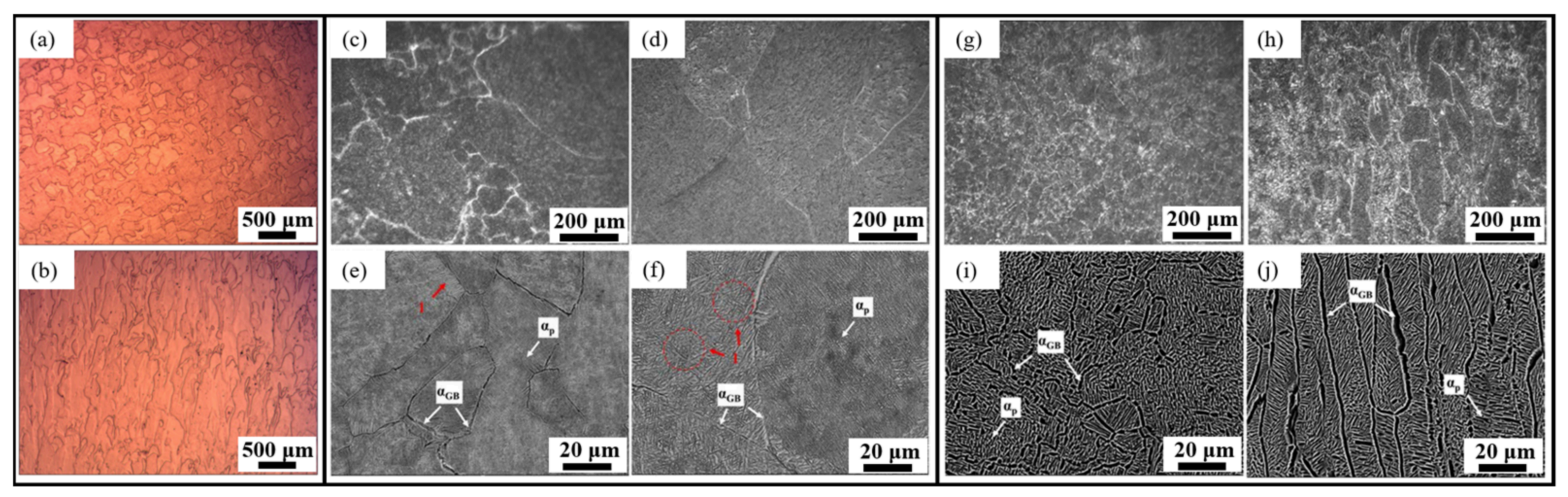
Microstructures of SLM-fabricated Ti-55531 titanium alloy and those after different HIP treatments: (**a**,**b**) as-SLM condition; (**c**–**f**) β-HIP condition; (**g**–**j**) α + β-HIP condition; (**a**,**c**,**e**,**g**,**i**) horizontal direction; (**b**,**d**,**f**,**h**,**j**) build direction. The red arrows indicate local α-phase precipitation, while the red dashed circles highlight clusters of acicular α precipitates. Reproduced from Ref. [47] with permission of the author.

Hot isostatic pressing (HIP) is a process in which powder is encapsulated in a container under a vacuum or protective atmosphere and placed in a high-pressure vessel, where the simultaneous application of elevated temperature and high pressure promotes the densification of powder particles, ultimately yielding a highly dense compact or component [45,46]. This technique effectively eliminates internal porosity and defects, substantially improving microstructural homogeneity and mechanical properties, and is particularly suitable for producing near-net-shape components with complex geometries. In the fabrication of Ti-55531 titanium alloy, HIP is frequently employed as a post-treatment step for additively manufactured parts, aiming to homogenize the microstructure and reduce defects, thereby enhancing the mechanical performance and service reliability of the components [23]. Zuo [47] investigated the influence of HIP on the microstructure and properties of SLM-fabricated Ti-55531 titanium alloy (results shown in Figure 9). HIP treatment in the β-phase field (880 °C/1 h/150 MPa) promoted the equiaxation of columnar β grains along the build direction and induced the precipitation of a fine lamellar α phase, significantly reducing microstructural anisotropy and increasing the ultimate tensile strength to approximately 1140 MPa while maintaining an elongation of about 12%. In contrast, HIP treatment in the α + β two-phase field (800 °C/2 h/150 MPa) preserved the columnar grain structure with coarser lamellar α precipitates, and the resulting mechanical properties exhibited pronounced anisotropy. Similar effects of microstructural control were also demonstrated by Cao et al. [48], who reported that HIP effectively reduced as-built defects and promoted microstructural evolution in additively manufactured Ti-55531 titanium alloy, thereby improving its mechanical properties.

**Figure 10:**
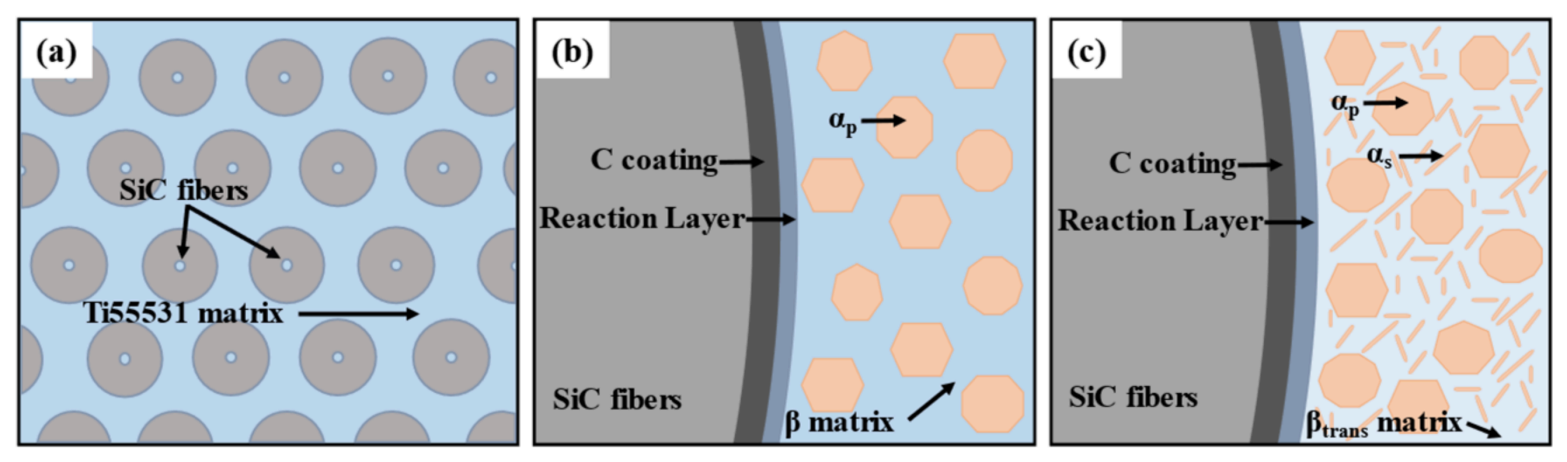
Schematic illustration of the microstructural evolution of SiC fiber-reinforced Ti55531 composites: (**a**) overall fiber distribution; (**b**) interfacial structure and matrix microstructure in the HIPed condition; (**c**) interfacial structure and matrix microstructure after heat treatment. Schematic illustration based on Ref. [49].

In addition to serving as a critical post-treatment for additively manufactured components, HIP technology is also widely applied in the fabrication and microstructure control of Ti-55531 titanium alloy-based composites. Li et al. [49] employed HIP to fabricate continuous SiC fiber-reinforced Ti-55531 composites and investigated the effects of heat treatment on the interfacial structure and mechanical properties (schematically illustrated in Figure 10). Their results reveal that the composite interface consists of SiC fibers, a C coating, a TiC reaction layer, and the Ti-55531 titanium alloy matrix, with a dense microstructure and good interfacial bonding. In the HIPed condition, the matrix mainly consists of equiaxed primary α and β phases. During heat treatment, fine acicular secondary α precipitates form within the β matrix, accompanied by a marked increase in texture intensity, which effectively impedes dislocation motion and enhances matrix strength, leading to a significant improvement in the tensile strength of the composite. Meanwhile, the interfacial reaction layer only undergoes slight thickening, exerting a limited influence on the overall performance.

Overall, the role of HIP in Ti-55531 titanium alloy can be broadly classified into two aspects. First, as a post-processing technique for additively manufactured components, HIP improves microstructural homogeneity and mechanical properties by reducing porosity and manufacturing defects and tailoring the β-grain structure and α-phase morphology. Second, HIP serves as an important densification and diffusion-bonding technique for Ti-55531-based composites, enabling effective bonding at both the fiber/matrix interfaces and internal interfaces within the matrix. However, HIP temperature, pressure, and holding time simultaneously influence phase transformations, grain evolution, and interfacial reactions. Therefore, further optimization of processing parameters is required for different initial microstructures and material systems.

## 3. Hot Deformation Behavior and Microstructural Evolution of Ti-55531 Titanium Alloy

As a near-β high-strength and high-toughness titanium alloy, Ti-55531 titanium alloy exhibits hot deformation behavior that is highly dependent on processing parameters. This behavior serves as the critical foundation for establishing rational thermomechanical processing routes and optimizing its microstructure and mechanical properties. Systematic investigations into its flow stress characteristics, microstructural evolution mechanisms, and processing windows during hot working are of great significance for optimizing forging and heat treatment procedures, ultimately facilitating the precise tailoring of the microstructure and properties.

### 3.1. Forging Windows and Microstructural Characteristics

The primary objective of forging is to obtain a microstructure that meets specific performance specifications. For titanium alloys, the morphology and volume fraction of the primary α phase, together with the prior-β grain size, are the key microstructural features governing mechanical properties; these features are primarily determined by the forging temperature parameters. The forging temperature range is defined relative to the T_β_. Accordingly, titanium alloy forging can be classified into four types: α + β, near-β, quasi-β, and β forging [50]. Each process yields distinct microstructural morphologies, such as lamellar, bimodal, or basket-weave structures.

#### 3.1.1. α + β Forging

α + β forging is typically conducted at temperatures 30–100 °C below T_β_, where both the α and β phases co-deform to accommodate the applied strain. With increasing deformation, the prior-β grains are first flattened, fragmented, and elongated along the deformation direction. Concurrently, the lamellar α phase undergoes distortion and fragmentation, also aligning along the primary deformation direction. Upon further deformation, pronounced banded structures begin to develop within the material. During this process, a fraction of the α phase undergoes dynamic recrystallization (DRX), transforming into fine equiaxed α grains, while the non-recrystallized α phase evolves into disk-shaped, rod-like, or fibrous morphologies. The fundamental mechanism of this process is the deformation-induced dynamic recrystallization of the α phase, which promotes its equiaxation and enables precise control over its size, morphology, and volume fraction, thereby achieving a microstructure that meets the targeted mechanical properties. Following α + β forging, the alloy exhibits relatively high ductility and room-temperature strength, albeit with comparatively lower high-temperature performance and fracture toughness [51].

#### 3.1.2. β Forging

β forging refers to a forging process conducted at temperatures approximately 50 °C or more above T_β_, which yields a basket-weave or Widmanstätten microstructure. The ingot breakdown stage for titanium alloys typically employs β forging, as high-temperature deformation facilitates the elimination or mitigation of casting defects and compositional segregation [52]. Owing to the elevated deformation temperature, β forging significantly enhances the workability of the alloy. The deformation resistance is substantially reduced, which minimizes energy consumption and die wear, thereby extending the die service life. Concurrently, the alloy’s material flow and hot-workability are enhanced, facilitating the forming of complex structural components and reducing the risk of defects such as cracking. Furthermore, β forging exerts a beneficial effect on properties such as creep strength, fracture toughness, and fatigue crack growth resistance. However, this process is highly sensitive to temperature control; improper control may induce β embrittlement, necessitating stringent regulation of temperatures during heating and die forging. In industrial practice, β forging is often combined with α + β forging to achieve an optimal balance between overall mechanical properties and processing compatibility.

#### 3.1.3. Near-β Forging

Near-β forging is a forging process in which the billet is heated to a temperature 10–15 °C below T_β_. Upon completion of deformation, rapid water quenching is typically applied, yielding a triplex microstructure composed of an equiaxed α phase, a lamellar α phase, and a transformed β matrix [53]. This microstructure combines the performance advantages of both equiaxed and basket-weave structures, delivering high ductility and good thermal stability, coupled with excellent fracture toughness and low-cycle fatigue resistance [54].

#### 3.1.4. Quasi-β Forging

Quasi-β forging is a specific forging process for titanium alloys, which proceeds as follows: the billet is first heated and held at a relatively low temperature 20–40 °C below T_β_, followed by a rapid increase in furnace temperature by 10–20 °C for a short duration, after which forging deformation commences immediately [55]. This process is designed to obtain a microstructure with a fine basket-weave morphology. Following quasi-β forging, the alloy exhibits high creep resistance, fracture toughness, and impact resistance, albeit with relatively lower ductility and thermal stability.

### 3.2. Heat Treatment Processes and Microstructural Tailoring

The mechanical properties of the Ti-55531 titanium alloy are highly dependent on the heat treatment procedures. The core objective of heat treatment is to achieve an optimal balance among strength, ductility, and toughness by controlling the morphology, size, and distribution of the α_p_ and secondary α (α_s_) phases. The heat treatments applicable to this alloy are numerous, primarily including solution treatment, aging treatment, and annealing.

#### 3.2.1. Solution Treatment

**Figure 11:**
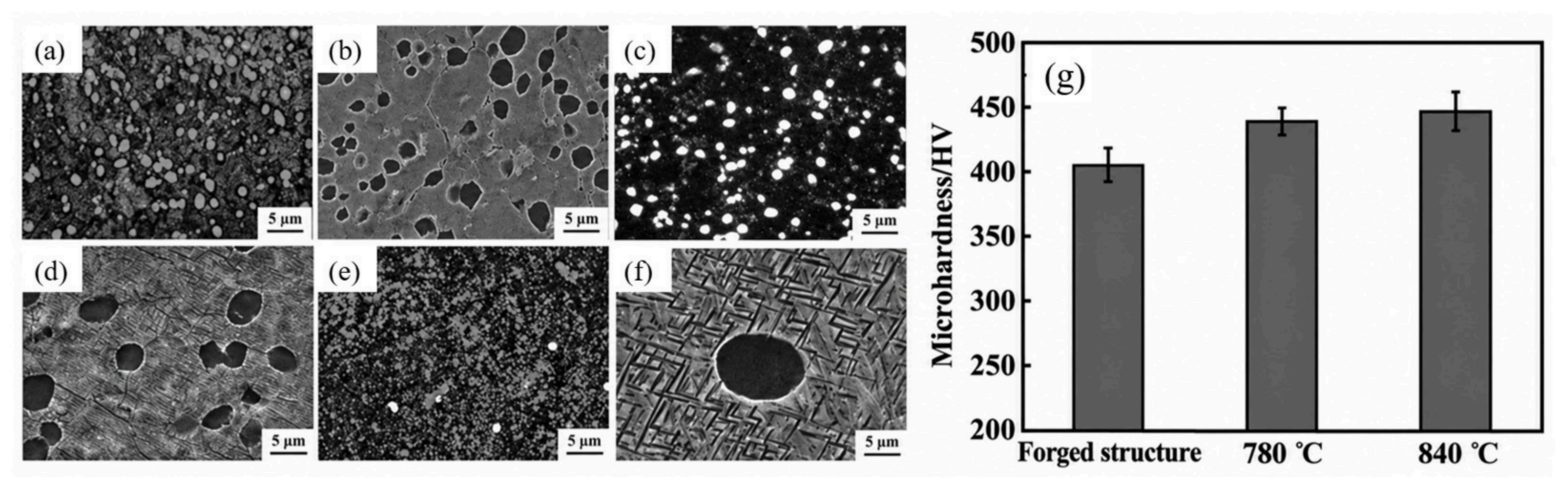
Optical microstructures (**a**,**c**,**e**), SEM images (**b**,**d**,**f**), and (**g**) microhardness of Ti-55531 before and after solution treatment. (**a**,**b**) As-received microstructure; (**c**,**d**) solution treatment at 780 °C + aging; (**e**,**f**) solution treatment at 840 °C + aging. Reproduced with permission from Ref. [56]. Copyright 2017, Transactions of Materials and Heat Treatment.

Solution treatment is typically conducted below the T_β_, generally within the temperature range of 790–830 °C. Following isothermal holding in this temperature range, water quenching is applied to obtain a supersaturated metastable β phase while retaining a certain fraction of the primary α phase. Studies have shown that with increasing solution temperature, the volume fraction of primary α decreases, leading to an increase in alloy strength but a corresponding reduction in ductility. Li et al. [56] investigated the effects of different solution temperatures on the microstructure and mechanical properties of forged Ti-55531 titanium alloy (results shown in Figure 11). The as-forged microstructure is primarily composed of globular α and β phases; after solution treatment at 780 °C or 840 °C followed by aging at 540 °C, the microstructure transforms into a duplex structure consisting of primary α, acicular secondary α, and a β matrix. With increasing solution temperature, the volume fraction of globular primary α markedly decreases, whereas the fraction of fine acicular secondary α increases, accompanied by an increase in hardness; the hardness increases by approximately 8.5% and 10.3% after solution treatment at 780 °C and 840 °C, respectively.

**Figure 12:**
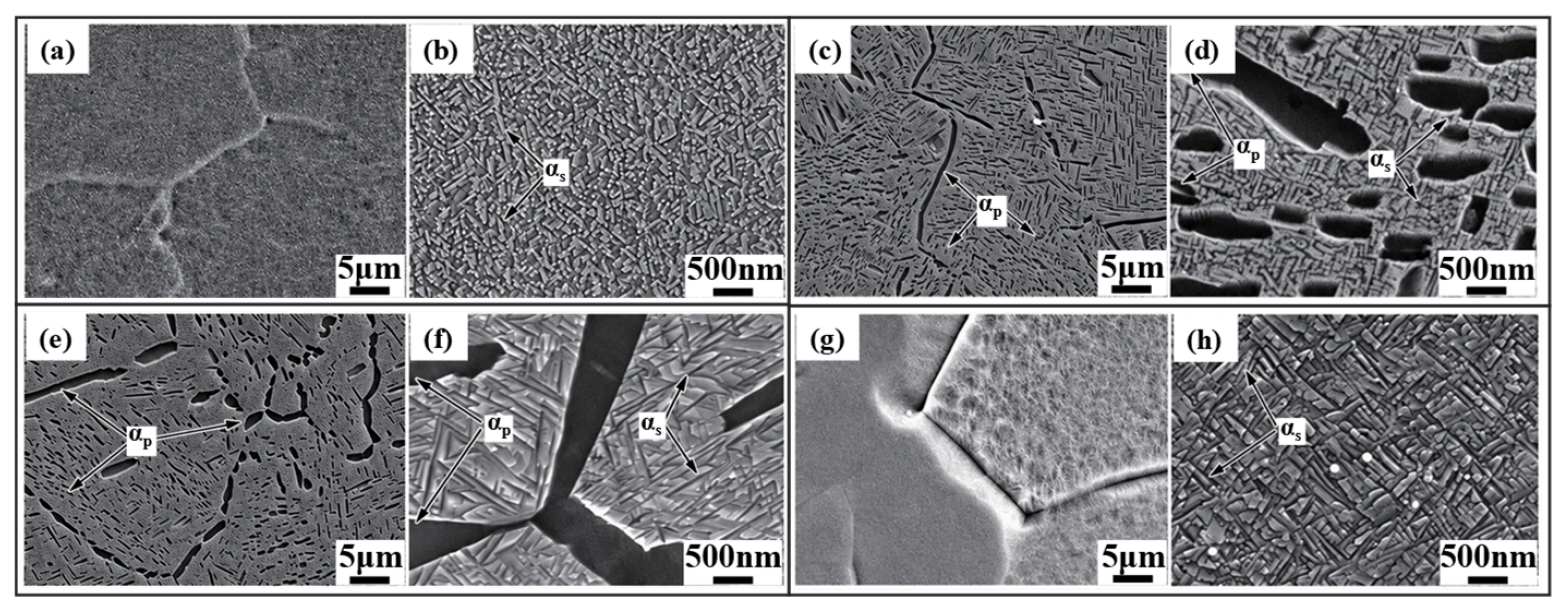
Low (**a**,**c**,**e**,**g**) and high (**b**,**d**,**f**,**h**) magnified SEM images of the porous Ti55531 specimens after solution treated at different temperatures for 1 h followed by aging at 500 °C for 4 h (α_p_—primary α, α_s_—secondary α): (**a**,**b**) without solution treatment; (**c**,**d**) 750 °C; (**e**,**f**) 800 °C; (**g**,**h**) 900 °C. Reproduced with permission from Ref. [43]. Copyright 2023, Acta Metallurgica Sinica.

A similar evolution trend has also been validated in additively manufactured Ti-55531 titanium alloy. Xu et al. [44] investigated the microstructure and mechanical properties of SLM-fabricated Ti-55531–0.5Nb at different solution temperatures. They found that with increasing solution temperature, the volume fraction of primary α gradually decreases while the precipitation of fine secondary α increases, resulting in a significant enhancement in strength. At a solution temperature of 790 °C, the alloy achieved an ultimate tensile strength of approximately 1300 MPa, albeit with a reduction in elongation to about 2.4%. This underscores the critical role of solution temperature in modulating the α-phase morphology and the strength–toughness balance. Furthermore, solution and aging heat treatments also enable effective tailoring of the α/β phase constitution in the solid matrix of the struts in SLM-fabricated Ti-55531 titanium alloy porous structures. Zhang et al. [43] examined the influence of solution treatment in the range of 750–900 °C and aging at 500–600 °C on porous Ti-55531 titanium alloy, revealing that increasing the solution temperature reduces the primary α content while increasing secondary α precipitation in the struts (see Figure 12). This enhances the strut matrix strength at the expense of ductility, which in turn influences the overall mechanical response of the porous architecture.

In summary, the solution temperature exerts a significant influence on the microstructure and mechanical properties of Ti-55531 titanium alloy by governing the dissolution of primary α and the precipitation behavior of secondary α. The underlying trend can be generalized as follows: higher solution temperatures lead to reduced primary α, increased precipitation of fine secondary α, enhanced strength, and decreased ductility.

#### 3.2.2. Aging Treatment

Aging is a critical method for regulating the microstructure and mechanical properties of Ti-55531 titanium alloy, with its strengthening effect primarily derived from the decomposition of the β phase and the precipitation behavior of α_s_ [57]. Studies have demonstrated that aging temperature and time significantly influence the morphology, size, and volume fraction of α_s_, thereby modulating the strength–ductility balance of the material.

**Figure 13:**
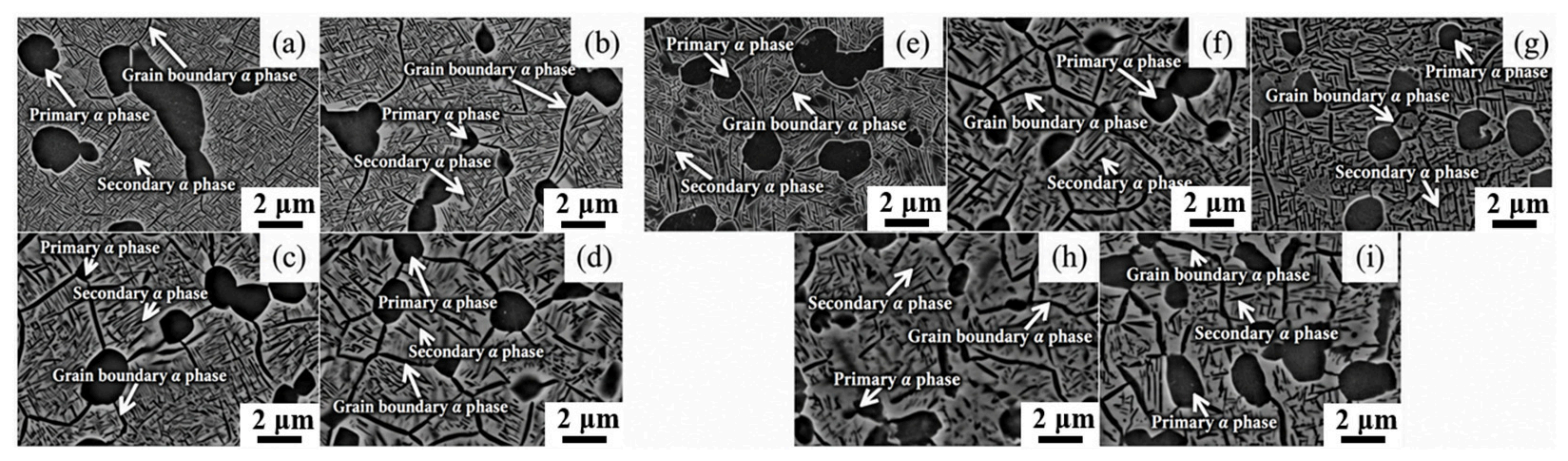
Microstructures of Ti-55531 titanium alloy after solution treatment at 800 °C for 2 h followed by different aging treatments: (**a**–**d**) aged for 7 h at 580, 600, 620, and 640 °C, respectively; and (**e**–**i**) aged at 640 °C for 6, 7, 8, 9, and 10 h, respectively. Reproduced with permission from Ref. [58]. Copyright 2022, Rare Metal Materials and Engineering.

Zhang et al. [58] investigated the microstructural evolution of Ti-55531 titanium alloy during aging at 580–640 °C following solution treatment at 800 °C. Their results indicate that fine lamellar α_s_ precipitates progressively from the β matrix during aging, and the α_s_ lamellae coarsen noticeably with increasing aging temperature and time, with the lamellar thickness growing at rates of approximately 1 nm/°C and 8 nm/h, respectively. After solution treatment at 800 °C/2 h followed by aging at 640 °C/8 h, the alloy achieved an optimal combination of mechanical properties, with an ultimate tensile strength of approximately 1144 MPa and an elongation of about 8.16%. Representative α_s_ morphologies and lamellar evolution are presented in Figure 13.

**Figure 14:**
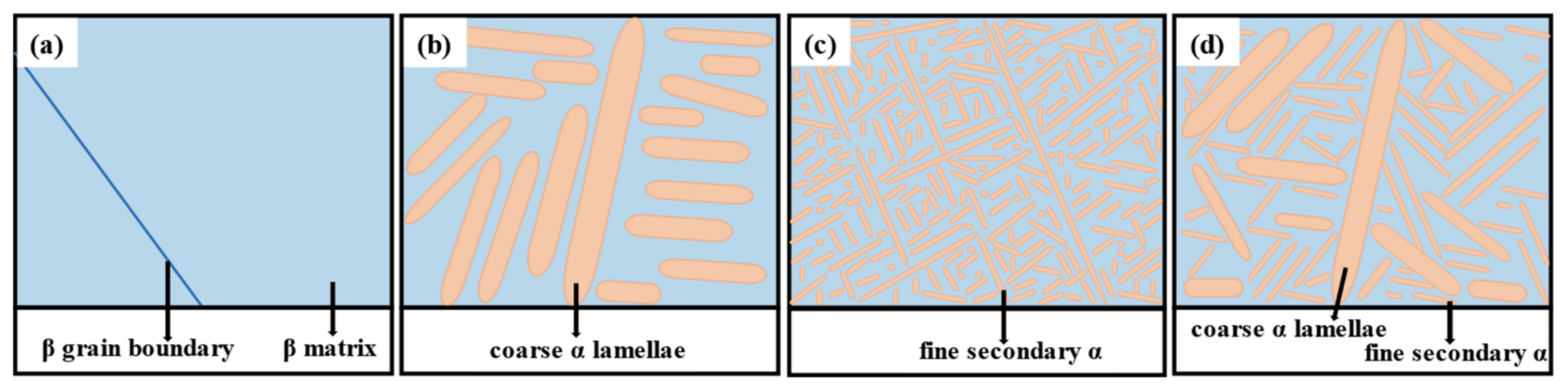
Schematic illustration of microstructural evolution in Ti55531 alloy during solution treatment and subsequent aging treatments: (**a**) solution treatment at 850 °C for 1 h; (**b**) subsequent aging at 700 °C for 3 h; (**c**) subsequent aging at 600 °C for 3 h; (**d**) two-step aging at 700 °C for 3 h followed by 600 °C for 3 h. Schematic illustration based on Ref. [17].

Building on this foundation, further studies have adopted two-step aging to engineer multi-scale α-phase structures. Wang et al. [17] employed a two-step aging regime at 700 °C/3 h + 600 °C/3 h to construct a multi-scale lamellar architecture composed of coarse α plates and fine α precipitates. The high-temperature stage promotes the nucleation and growth of α lamellae, while the low-temperature stage induces the formation of fine dispersed α particles via a spinodal-decomposition-like mechanism, thereby creating a high-density α/β interfacial structure. This multi-scale lamellar configuration effectively elevates the resistance to dislocation slip, yielding an exceptional strength-ductility synergy with a yield strength of ~1.1 GPa and an elongation of ~19.5%, representing a marked improvement in the strength–ductility synergy. The microstructural evolution under different heat treatment conditions is schematically illustrated in Figure 14.

In addition to room-temperature properties, aging precipitates also exert a notable influence on high-temperature service performance. Ye et al. [23] fabricated Ti-55531 titanium alloy via a hybrid additive manufacturing and forging route and conducted aging treatments at 400–500 °C. Their findings reveal that the fine α precipitates formed after aging significantly enhance the microstructural stability at elevated temperatures, achieving a tensile strength of 997 MPa, a yield strength of 878 MPa, and an elongation of approximately 11% at 400 °C, underscoring the crucial role of α precipitation in improving the high-temperature mechanical performance of Ti-55531 titanium alloy.

Furthermore, alloying additions and composite architectures also significantly affect the aging precipitation behavior. Qi et al. [27] investigated the influence of rare-earth La addition and heat treatment on the microstructural evolution of Ti-55531 titanium alloy, demonstrating that La refines the as-cast microstructure and promotes uniform α precipitation during aging, thereby improving the strength–ductility balance. Concurrently, in the SiC_f_/Ti-55531 composite system, prolonged low-temperature aging promotes the precipitation of fine acicular α_s_ within the β matrix, forming a high-density α_s_/β interfacial network. These interfaces effectively hinder dislocation motion and reinforce the matrix strength while enhancing the texture intensity along the fiber direction, further boosting the tensile performance of the composite [49].

In summary, the property enhancement of Ti-55531 titanium alloy during aging is primarily governed by the decomposition of the β phase and the precipitation-strengthening mechanism of α_s_. Appropriate aging schedules enable the formation of fine and dispersed α precipitates combined with a high density of α/β interfaces, leading to a substantial increase in strength. However, excessive coarsening or overly high volume fractions of α may compromise ductility. Therefore, the judicious design of aging temperature and time to tailor the morphology and scale of α precipitates is the key to achieving the synergistic optimization of strength and ductility in Ti-55531 titanium alloy.

#### 3.2.3. Annealing Treatment

Annealing is an essential process for regulating the microstructural stability and stress state of the near-β Ti-55531 titanium alloy. Its primary functions are to eliminate crystal defects and residual stresses introduced during thermomechanical processing, while simultaneously optimizing the grain structure through recovery and recrystallization [52], thereby improving the overall mechanical properties of the material.

**Figure 15:**
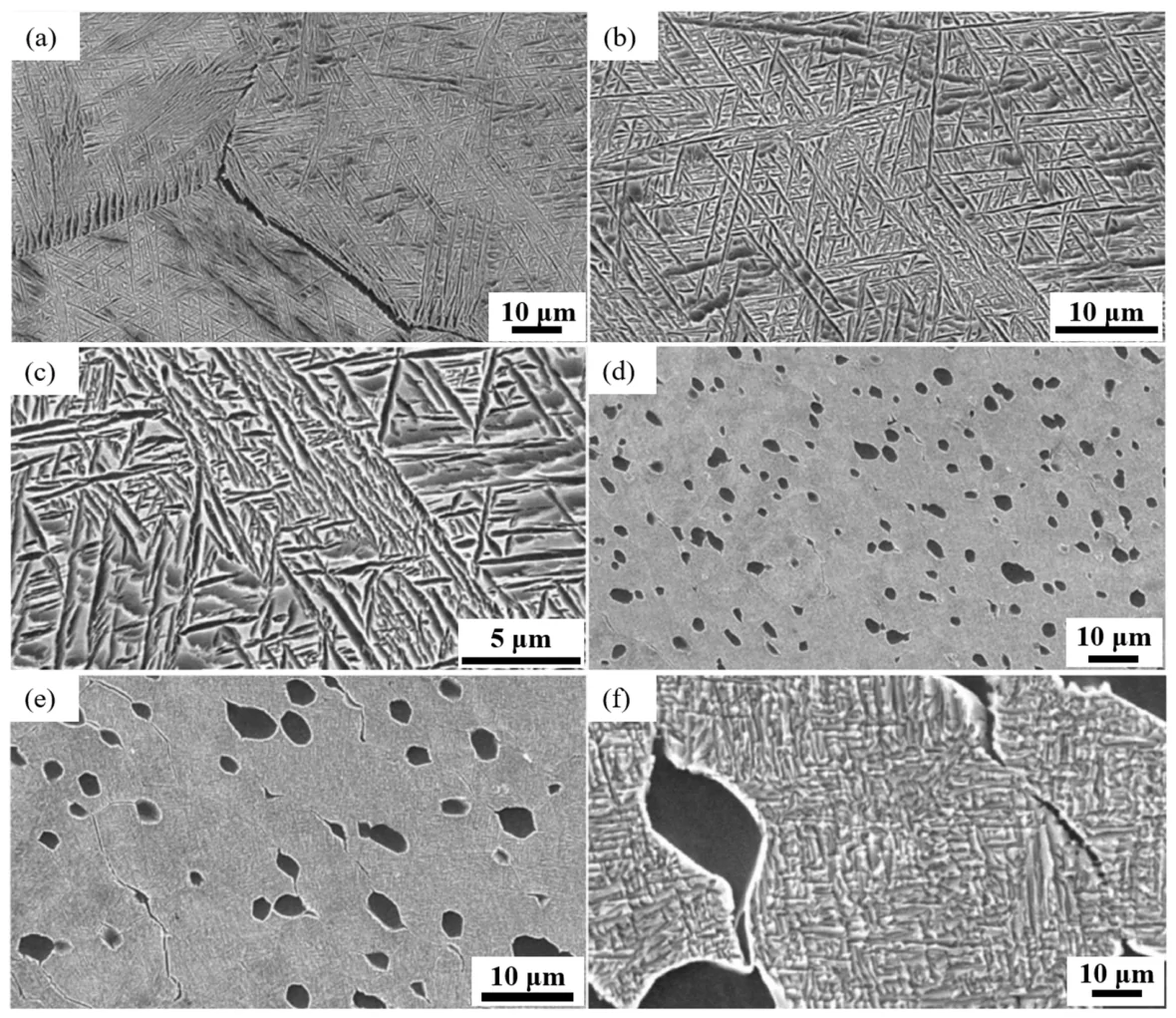
SEM images of Ti-55531 titanium alloy after β annealing (**a**–**c**) and solution strengthening (**d**–**f**). Reproduced from Ref. [59] with permission of the author.

Various annealing schedules also exert notable influences on the microstructure and properties of Ti-55531 titanium alloy. Gao et al. [59] investigated the effects of two typical heat-treatment regimens—β annealing and solution strengthening—on the microstructure and mechanical properties of Ti-55531 titanium alloy (Figure 15, Table 1). Their results indicate that after β annealing, the alloy develops a relatively fine lamellar α-phase microstructure, achieving a favorable balance between strength and ductility, with an ultimate tensile strength of approximately 1170 MPa, an elongation of about 11%, and a fracture toughness of 97.6 MPa·m^1/2^. In contrast, although the solution-strengthening treatment could increase the tensile strength to approximately 1380 MPa, it resulted in a notable degradation of ductility and fracture toughness. These findings demonstrate that annealing treatments can enhance both toughness and overall mechanical performance while maintaining a relatively high strength level.

**Figure 16:**
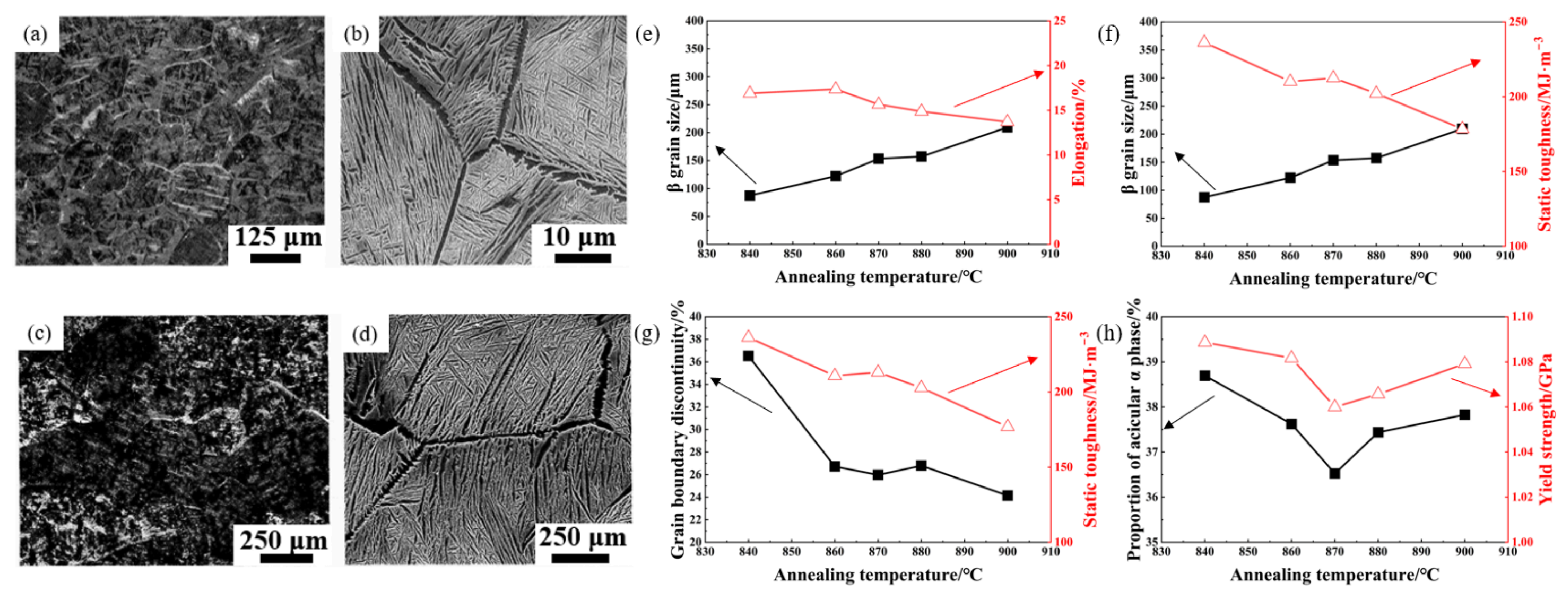
Microstructures and microstructure–property relationships of annealed Ti-55531 titanium alloy: (**a**,**b**) OM and SEM images at 840 °C; (**c**,**d**) OM and SEM images at 900 °C; (**e**,**f**) β-grain size versus elongation and static toughness, respectively; (**g**) grain-boundary α discontinuity versus static toughness; (**h**) acicular α fraction versus yield strength. Reproduced with permission from Ref. [60]. Copyright 2022, Aerospace Materials & Technology.

Based on the optimized heat treatment regime, elucidating the correlation between annealing parameters and microstructural evolution is crucial for understanding the underlying mechanisms governing the changes in alloy mechanical properties. Li et al. [60] further investigated the effect of β-phase region annealing temperature on the microstructure and mechanical properties of the Ti-55531 titanium alloy, with the results presented in Figure 16. It was revealed that with increasing annealing temperature, the β grains underwent marked growth, the intragranular α lamellae coarsened, and the continuity of the α_GB_ phase diminished, whereas the fraction of acicular α exhibited a trend of initially decreasing and subsequently increasing. The results demonstrate that the β grain size, the continuity of the α_GB_, and the volume fraction of the α phase collectively govern the mechanical properties. Specifically, the degree of discontinuity of the α_GB_ is positively correlated with toughness, while an increased fraction of the α phase contributes to an improvement in yield strength.

During the forging process, the material undergoes hot deformation, and the deformation-induced dislocations, subgrain structures, and stored energy significantly influence the restoration behavior of the microstructure during subsequent annealing. Consequently, the microstructural evolution during post-deformation annealing has also garnered considerable attention. Cai [61] examined the influence of isothermal compression deformation and subsequent heat treatment on the microstructural evolution of the Ti-55531 titanium alloy. They found that the subgrain and dislocation structures generated during hot deformation promote static recrystallization (SRX) of the β phase during annealing, which progressively eliminates the deformed microstructure and facilitates grain refinement. Simultaneously, the morphology of the post-deformation α phase is modified, and the microstructural features at the α/β interfaces exert a significant influence on the nucleation behavior of β recrystallization.

In summary, heat treatment plays a critical role in the microstructural regulation and performance optimization of the Ti-55531 titanium alloy. Solution treatment primarily governs the stability of the β phase and the morphology of primary α, establishing the foundation for subsequent microstructural evolution. Aging treatment achieves significant precipitation strengthening through the decomposition of the β phase and the precipitation of fine secondary α. Annealing promotes dislocation recovery, recrystallization, and the stabilization of lamellar α structures, thereby imparting a more homogeneous and stable microstructure to Ti-55531 titanium alloy and enabling a coordinated optimization of strength, ductility, and fracture toughness. These three processes act in concert to collectively determine the final microstructure and mechanical properties of the alloy. Therefore, the rational design of solution, aging, and annealing schedules enables the synergistic optimization of strength, ductility, and damage tolerance performance in Ti-55531 titanium alloy, providing a vital microstructural engineering tool for its application in high-performance aerospace structures.

### 3.3. Hot Deformation Mechanisms and Modeling Approaches

Thermomechanical simulators are the primary experimental tools for investigating the high-temperature deformation behavior of the Ti-55531 titanium alloy, with common test modes including uniaxial tension, uniaxial compression, and torsion. Based on thermomechanical simulation data, the flow behavior of the material during high-temperature deformation can be systematically analyzed. Investigation of the high-temperature deformation behavior of Ti-55531 titanium alloy is fundamental to the development of rational thermomechanical processing routes, providing a reliable theoretical basis for process parameter optimization. Current research priorities in this field mainly encompass the establishment of constitutive models, analysis of microstructural evolution patterns, and the prediction of instability and stability domains based on processing maps. Modern hot-deformation research has progressively shifted from traditional trial-and-error approaches to precise, model-driven designs, with constitutive equations and processing maps serving as the two core theoretical tools.

#### 3.3.1. Constitutive Equations

**Figure 17:**
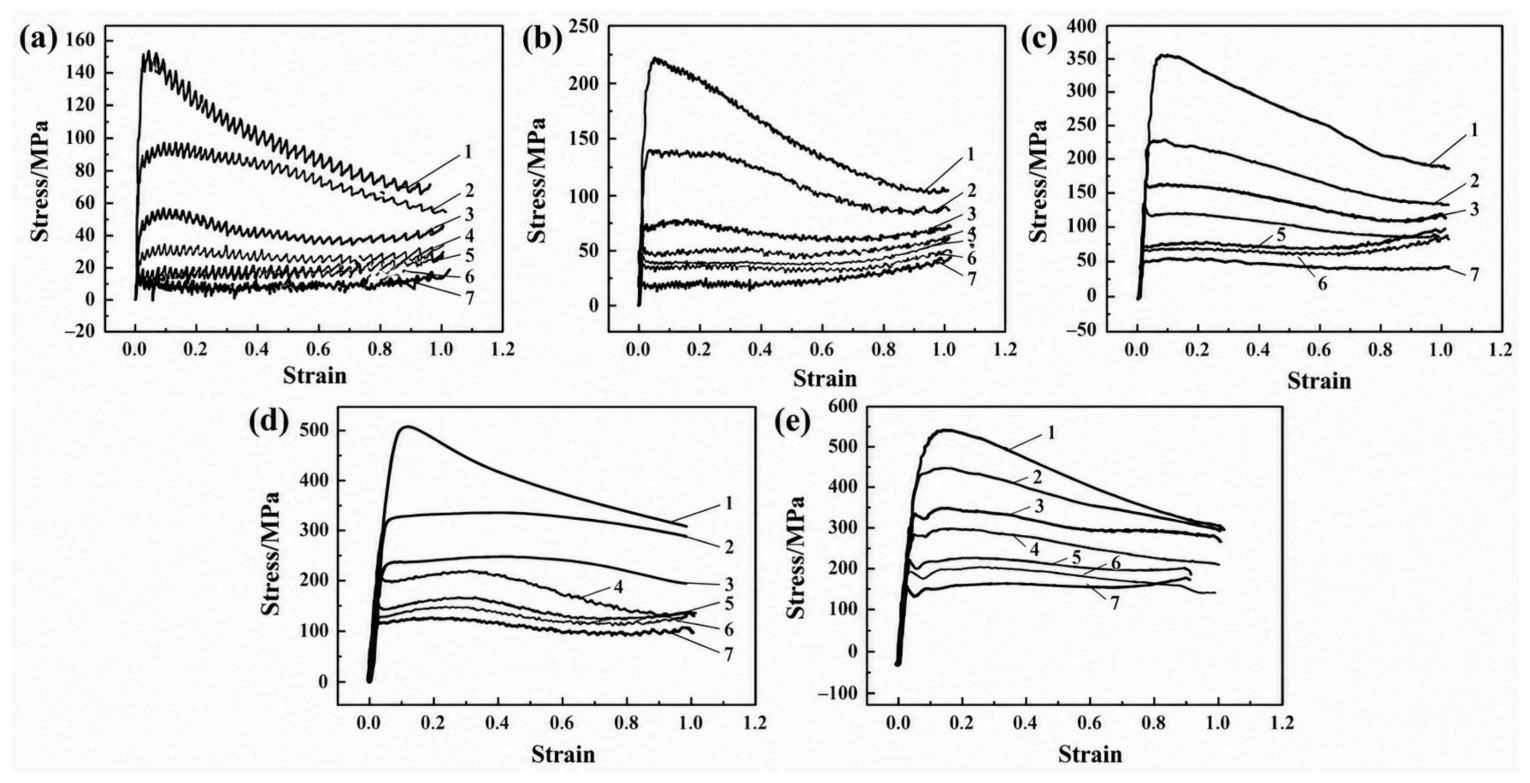
True stress–true strain curves of the titanium alloy under different deformation conditions: (**a**) 0.001 s^−1^, (**b**) 0.01 s^−1^, (**c**) 0.1 s^−1^, (**d**) 1 s^−1^, (**e**) 10 s^−1^. The corresponding deformation temperatures are indicated by numbers: (1) 700 °C, (2) 750 °C, (3) 800 °C, (4) 850 °C, (5) 900 °C, (6) 950 °C, (7) 1000 °C. Reproduced with permission from Ref. [64]. Copyright 2015, Forging & Stamping Technology.

The constitutive relationship for hot deformation of the Ti-55531 titanium alloy is typically described using the Arrhenius-type hyperbolic sine equation, which is capable of providing reliable predictions of flow stress under various hot-working conditions [62]. Li et al. [63] investigated the deformation behavior of the alloy in the temperature range of 700–1000 °C and strain rate range of 0.001–10 s^−1^, and found that the true stress–true strain curves exhibit dynamic recrystallization characteristics during deformation in the α + β two-phase region, whereas dynamic recovery features predominate in the β single-phase region. The stress–strain curves at different temperatures and strain rates are presented in Figure 17, showing that flow stress decreases with increasing temperature and increases with increasing strain rate. On this basis, Arrhenius-type constitutive equations were established separately for the two-phase and single-phase regions, with relative errors between predicted and experimental values below 10%, adequately reflecting the hot-deformation flow behavior of the alloy. Yang et al. [64] conducted uniaxial hot-compression tests on a fully β-solutionized Ti-55531 titanium alloy at seven temperatures spanning the α + β two-phase region, the β region, and temperatures near the transus, at three strain rates (0.01, 0.1, and 1 s^−1^), establishing a reasonable spinning temperature range of 650–800 °C and constructing a constitutive equation for deformation in the α + β region based on the hyperbolic sine model, thereby providing a theoretical basis for subsequent finite-element simulations and hot-spinning process design.

**Figure 18:**
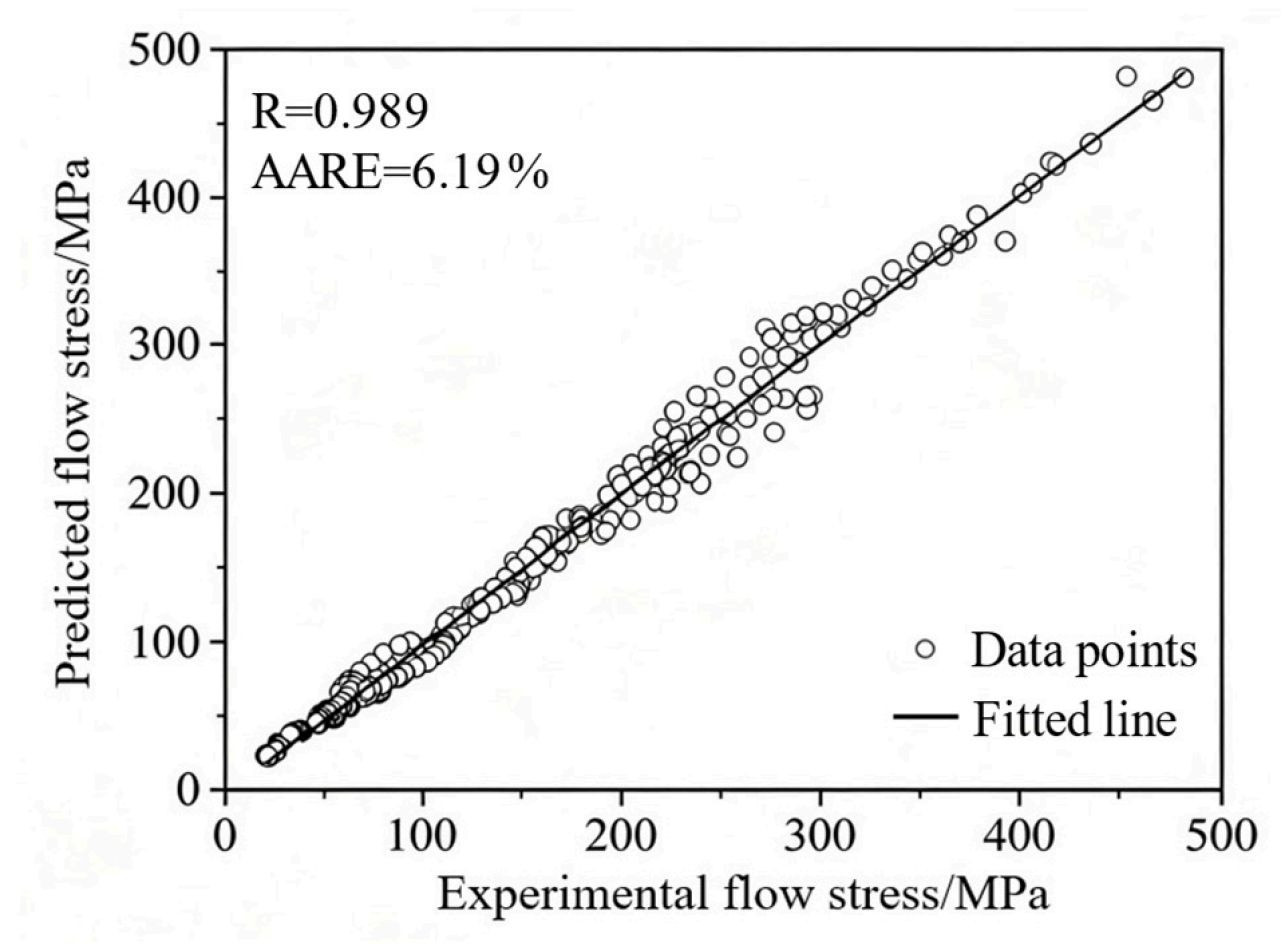
Comparison between predicted and experimental values. Reproduced from Ref. [24] with permission of the author.

In terms of model refinement, Han [24] introduced a strain-compensation mechanism to establish a strain-dependent Arrhenius constitutive model for predicting the flow stress of Ti-55531 titanium alloy in both the two-phase and single-phase regions. A comparison between predicted and experimental values is shown in Figure 18, demonstrating excellent agreement with a correlation coefficient of 0.989. Wu et al. [65] investigated the deformation behavior in the β single-phase region (860–900 °C, 0.001–10 s^−1^) and found that the true stress–true strain curves exhibit pronounced dynamic recrystallization characteristics, accompanied by discontinuous yielding and flow softening. The constitutive equation constructed on the basis of the hyperbolic sine function yielded a correlation coefficient exceeding 0.98 and an average absolute relative error below 5.7%, indicating good predictive accuracy. In the α + β two-phase region (730–820 °C), Wu et al. [66] further established a strain-compensated Arrhenius-type constitutive equation, with predicted stresses showing excellent agreement with experimental data, achieving a correlation coefficient of 0.99 and a maximum average absolute relative error of 7.94%. Zheng et al. [67] also established a strain-compensated constitutive equation in the temperature range of 730–900 °C and strain rate range of 0.001–10 s^−1^, achieving an average relative error of 7.44% in predicting the flow stress of Ti-55531 titanium alloy, further validating the effectiveness of the strain-compensation approach in describing the hot-deformation behavior of this alloy. In addition to Arrhenius-type constitutive models, Shokry et al. [68] developed modified Fields–Backofen and Zerilli–Armstrong models based on the hot deformation data of Ti-55531 titanium alloy. Among these, the modified Fields–Backofen model exhibited good agreement between the predicted and experimental results, achieving a correlation coefficient of 0.996 and an AARE of 3.34%. In recent years, machine learning approaches have also been increasingly employed in constitutive modeling of Ti-55531 titanium alloy. Hu et al. [69] compared a conventional Arrhenius-type model with a backpropagation (BP) neural network optimized using a swarm intelligence algorithm. Their results demonstrated that the AHA-BP model exhibited high predictive accuracy under previously unseen strain-rate conditions, providing a promising approach for improving the accuracy of flow stress prediction in Ti-55531 titanium alloy.

Collectively, existing studies indicate that the Arrhenius-type hyperbolic sine model is widely employed to describe the hot deformation behavior of Ti-55531 titanium alloy, although differences remain in its applicable conditions and parameter determination methods. In recent years, the introduction of modified phenomenological models and machine learning approaches has further expanded the available strategies for predicting the flow stress of this alloy. Early investigations generally established separate constitutive relationships for different phase fields, whereas subsequent studies increasingly incorporated strain compensation to account for the evolution of material parameters with increasing strain. Most existing models have achieved high correlation coefficients and low prediction errors, demonstrating their ability to adequately describe the flow stress response of Ti-55531 titanium alloy under the corresponding experimental conditions. A comparison of the constitutive models developed for Ti-55531 titanium alloy under different deformation conditions, together with their prediction accuracy, is presented in Table 2. However, differences in initial microstructure, deformation temperature range, strain rate, and phase field among individual studies preclude a rigorous direct comparison of their predictive accuracy metrics. Furthermore, Arrhenius-type models primarily establish empirical relationships between hot working parameters and flow stress, offering limited direct insight into microstructural evolution processes such as dynamic recovery, dynamic recrystallization, and phase transformation. Therefore, integrating flow stress constitutive models with dynamic recrystallization kinetics and microstructural evolution models represents an important direction for improving the predictive capability of models for complex hot working processes of Ti-55531 titanium alloy.

#### 3.3.2. Processing Maps

Processing maps are important analytical tools for the optimization of hot metal-forming processes. They can be used to predict microstructural changes during deformation, analyze high-temperature deformation mechanisms under different conditions, and thereby determine optimal deformation parameters while avoiding defect formation, thus providing a theoretical basis for thermomechanical processing [70].

**Figure 19:**
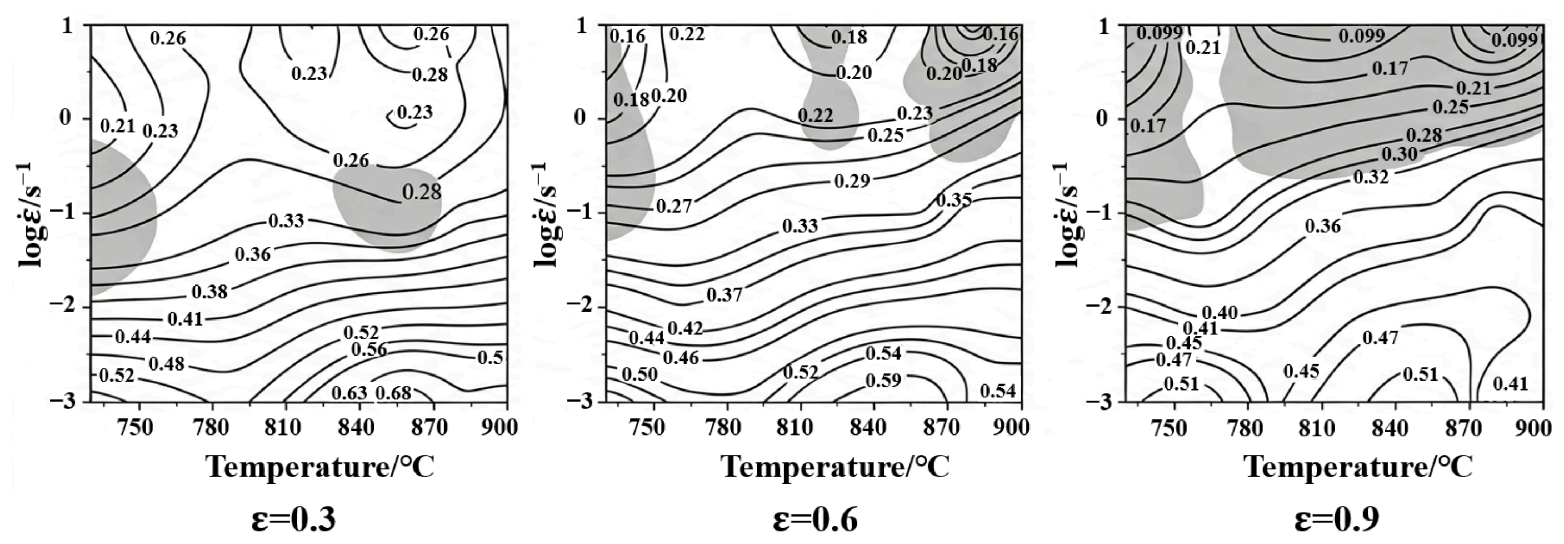
Processing maps of the Ti-55531 titanium alloy. Reproduced from Ref. [24] with permission of the author.

Through processing maps, the high-temperature plastic deformation mechanisms of materials under various deformation conditions can be analyzed, and safe and instability domains can be identified, enabling the design of rational thermomechanical processing routes for microstructural stability control, defect prevention, and yield improvement. In recent years, the application of processing map methodology to the study of hot-deformation behavior in titanium alloys has become increasingly prevalent. Han [24] investigated the hot-working behavior of Ti-55531 titanium alloy over the temperature range of 730–900 °C and strain rate range of 0.001–10 s^−1^, and constructed processing maps at true strains of 0.3, 0.6, and 0.9 (Figure 19). The results indicate that the power dissipation efficiency factor η increases with decreasing strain rate, reaching a peak in the range of 0.001–0.003 s^−1^ and 820–880 °C, which corresponds to favorable hot-workability. The instability domain is primarily concentrated in the low-temperature, high-strain-rate region and expands with increasing strain. The study identified the optimal hot-deformation processing window as: strain rate 0.001–0.01 s^−1^ and temperature 820–880 °C. Microstructural analysis reveals that the instability domain is characterized by localized plastic flow, whereas the stability domain exhibits dynamic recrystallization with significant grain refinement, which is consistent with the processing map predictions, thereby providing a basis for optimizing the hot-forming processes of this alloy.

Building on these findings, Wu et al. [65] further investigated the hot working behavior of Ti-55531 titanium alloy in the single-phase β region over a temperature range of 860–900 °C and strain rates of 0.001–10 s^−1^. Their results revealed that the severity of flow instability increased with increasing strain, while higher deformation temperatures and lower strain rates favored dynamic recrystallization of the β phase. Compared with the study by Han et al., which covered a broader temperature range, Wu et al. focused exclusively on the single-phase β region, further highlighting that the processing maps of Ti-55531 titanium alloy are governed not only by deformation temperature and strain rate but also by the phase field in which deformation occurs. Furthermore, Dikovits et al. [71] investigated the hot deformation behavior of Ti-55531 titanium alloy near the T_β_. By comparing different instability criteria and correlating their predictions with OM and EBSD analyses, they identified discrepancies between the instability regions predicted by different models and the experimentally observed microstructures. These findings indicate that processing map predictions should be comprehensively evaluated in conjunction with microstructural characteristics.

Processing maps constructed over different experimental parameter ranges also exhibit notable differences. Yang et al. [72] conducted hot compression experiments on forged Ti-55531 titanium alloy at temperatures of 790–940 °C, strain rates of 0.01–1 s^−1^, and a compression reduction of 60%, and subsequently constructed processing maps at a true strain of 0.6. Unlike Han [24], who identified distinct instability regions within a strain-rate range extending to 10 s^−1^, Yang et al. reported positive values of the flow instability parameter throughout their investigated processing conditions, with no flow instability region identified. It should be noted that the strain-rate ranges examined in these two studies differed considerably: the former extended to 10 s^−1^, whereas the latter was limited to 1 s^−1^. Therefore, their findings should not be regarded as inherently contradictory. This comparison highlights the strong dependence of the identified instability regions on the selected experimental parameter range. If the investigated conditions do not encompass sufficiently high strain rates, certain potential flow instability behaviors may not be captured in the resulting processing maps.

Beyond uniaxial hot compression, Zhu et al. [73] investigated the hot working behavior and microstructural evolution of Ti-55531 titanium alloy during multidirectional forging in the α + β two-phase region based on the dynamic materials model (DMM). Their results indicated that relatively high-power dissipation efficiencies (η > 0.3) could be achieved at deformation temperatures of 780–820 °C and strain rates of 0.01–0.36 s^−1^. Further multidirectional forging experiments revealed that increasing the deformation temperature increased the fraction of high-angle grain boundaries in the β matrix and promoted grain refinement, while accumulated deformation further reduced the β-grain size. These findings demonstrate that regions of high-power dissipation efficiency identified from processing maps must be validated against actual microstructural evolution to determine whether they are genuinely favorable for grain refinement and microstructural homogenization. In recent years, data-driven approaches have also been increasingly introduced into the construction of processing maps for Ti-55531 titanium alloy. Hu et al. [69] integrated conventional phenomenological constitutive models with machine learning methods to construct processing maps at different strains using predicted flow stress data. The processing regions were further evaluated in conjunction with activation energy maps and microstructural analyses. Their results revealed that, at a true strain of 0.8, the favorable processing window was located at 857–890 °C and 0.0125–0.0372 s^−1^, whereas flow instability was primarily concentrated within the intermediate strain-rate range of 0.1–1 s^−1^. These findings provide an alternative approach for optimizing the hot working parameters of Ti-55531 titanium alloy.

Collectively, existing studies suggest that relatively favorable hot working conditions for Ti-55531 titanium alloy generally lie within intermediate-to-high temperature ranges and low-to-intermediate strain rates, whereas flow instability is more likely to occur at lower temperatures and higher strain rates. Nevertheless, the specific processing windows and instability regions reported in different studies are not entirely consistent. These discrepancies are closely associated with variations in initial microstructure, T_β_, deformation phase field, strain, and the investigated temperature and strain-rate ranges. Therefore, processing windows reported in different studies should not be directly compared solely on the basis of absolute deformation temperatures and strain rates. Instead, a comprehensive assessment should account for the initial material condition and the deformation temperature relative to the corresponding T_β_. Furthermore, a high power dissipation efficiency does not necessarily correspond to an optimal hot-worked microstructure. Processing map predictions should therefore be validated through microstructural characterization, particularly with respect to dynamic recovery, dynamic recrystallization, grain refinement, and strain localization [74]. To facilitate a comparison of existing studies, Table 3 summarizes the deformation conditions, favorable processing regions, and flow instability characteristics reported for Ti-55531 titanium alloy under different processing-map approaches.

#### 3.3.3. Numerical Simulation

Finite-element modeling (FEM) has been widely applied in the fields of mechanical engineering, materials science, automotive engineering, and aerospace, and has become an important analytical tool in the study of plastic forming [75]. In the domain of metal plastic forming, this technique is primarily used for defect prediction and optimization during the design stages of forming processes and tooling, enabling the early identification of irrationalities and thereby reducing the number of trial productions and die modifications, thus lowering R&D costs. Furthermore, FEM provides visualization of the forming process, intuitively revealing material flow and deformation behavior. Forging is an important production route for titanium alloy components. A well-designed forging process not only yields parts with specific geometries but also enables the synergistic optimization of microstructure and mechanical properties. FEM can accurately present multiple field variables during hot forming, including temperature, stress, strain, and microstructural distributions. The integration of this technique into forging process design for analyzing plastic deformation and microstructural evolution can significantly enhance the rationality of process design [76].

Lv et al. [77] developed a Johnson–Mehl–Avrami–Kolmogorov (JMAK)-type DRX kinetics model for Ti-55531 titanium alloy based on hot compression experiments. The model was implemented into the DEFORM-3D finite element software through a user-defined FORTRAN subroutine to simulate hot compression under different deformation temperatures and strain rates. The results revealed a pronounced heterogeneous distribution of the DRX volume fraction within the compressed specimens, with a higher degree of recrystallization in the central region and a lower degree near the specimen ends in contact with the compression platens. The overall DRX volume fraction increased with increasing deformation temperature or decreasing strain rate. The simulated DRX volume fractions agreed well with the experimental measurements, demonstrating that coupling recrystallization kinetics with finite element analysis enables the prediction of microstructural heterogeneity during hot deformation of Ti-55531 titanium alloy.

Wu et al. [65] integrated the established high-temperature deformation constitutive model and dynamic recrystallization kinetics model into a user-defined subroutine of the DEFORM software, and conducted finite-element simulation of the forging process for Ti-55531 titanium alloy cylindrical specimens under non-isothermal conditions. In the simulation, the initial workpiece temperature was set at 890 °C, the die temperature at 100 °C, the strain rate at 0.1 s^−1^, and the compression was carried out to a height reduction of 60%. An axisymmetric model was adopted with consideration of the coupling between temperature and microstructural fields, and the calculated load–stroke curves showed good agreement with experimental measurements. Metallographic comparison confirms that the simulated recrystallization microstructural features are consistent with experimental results, demonstrating that the integrated model can effectively predict the macroscopic deformation and microstructural evolution of Ti-55531 titanium alloy during non-isothermal forging, thereby providing a reliable numerical simulation tool for actual process parameter optimization. Compared with Lv et al., who primarily validated their model against the DRX volume fraction measured in hot-compressed specimens, Wu et al. further extended their model to the non-isothermal forging of larger workpieces, enabling the simultaneous prediction of material flow behavior and microstructural evolution.

Zheng et al. [67] performed finite-element simulation of the hot-compression process for Ti-55531 titanium alloy at 860 °C and 0.1 s^−1^ to validate the established dynamic recrystallization model. The simulation results show that the average dynamically recrystallized grain size in the severe deformation zone is approximately 2.4 μm, representing a marked refinement compared to the initial grain size of about 5 μm; the grain size in the free deformation zone is approximately 3 μm with relatively minor refinement; whereas the difficult-to-deform zone, where the strain does not reach the critical value, exhibits almost no change in grain size. Comparison of the simulated grain size distribution with metallographic observations shows good consistency, confirming that the dynamic recrystallization model can accurately predict the microstructural evolution of Ti-55531 titanium alloy during hot deformation, in a separate study, Zheng [75] further applied finite element simulation to the optimization of the forging process for a Ti-55531 titanium alloy torque arm. Potential forming defects were identified through numerical simulation, and the forming process was optimized through preform design, the adoption of a two-stage forging process involving pre-forging and final forging, and modifications to the die geometry.

Collectively, existing studies indicate that finite element modeling of Ti-55531 titanium alloy has progressively evolved from the validation of DRX models at the hot-compression specimen scale towards coupled thermo-mechanical–microstructural predictions under non-isothermal conditions and the optimization of forming processes for complex components. Current models can adequately capture variations in the DRX volume fraction and grain size arising from heterogeneous distributions of temperature, strain, and strain rate, thereby providing an important link between the fundamental hot deformation behavior of the alloy and practical forging processes. Nevertheless, most existing models are still calibrated using uniaxial hot compression data and primarily employ the overall DRX volume fraction and average grain size as microstructural state variables. The competition among DRV, CDRX, and DDRX, as well as the coupling between α-phase precipitation and β-phase recrystallization during deformation across different phase fields, remains insufficiently accounted for. Moreover, boundary conditions such as friction, interfacial heat transfer, and the initial microstructure can affect the transferability of simulation results to industrial-scale, multi-pass forging processes. Future studies should therefore further integrate phase transformation kinetics, the distinct recrystallization mechanisms, and multiscale microstructural evolution models into finite element frameworks, while strengthening the quantitative validation of local microstructural predictions through experimental characterization techniques such as EBSD [78]. To facilitate a comparison of the existing studies, Table 4 summarizes the simulation objects, deformation conditions, constitutive and microstructural models, finite element approaches, and main predicted results reported for Ti-55531 titanium alloy.

## 4. Microstructural Evolution Mechanisms During Hot Deformation

**Figure 20:**
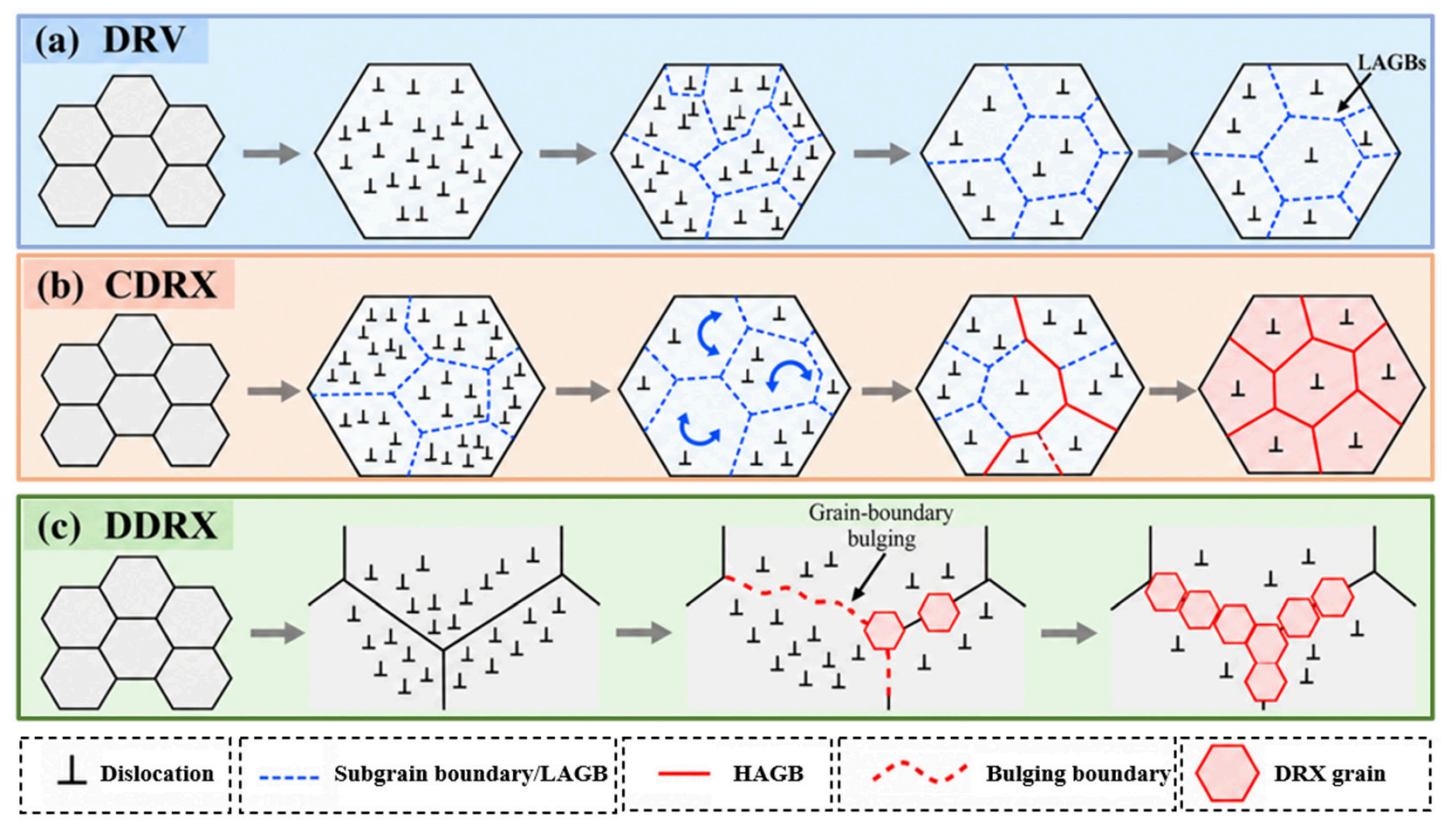
Schematic illustration of the dynamic recovery and dynamic recrystallization mechanisms during hot deformation of Ti-55531 titanium alloy: (**a**) DRV; (**b**) CDRX; and (**c**) DDRX.

Microstructural evolution is a fundamental phenomenon during the hot deformation of metallic materials. Owing to variations in initial microstructures and deformation parameters, the Ti-55531 titanium alloy typically exhibits multiple deformation mechanisms during hot working. The processing parameters significantly influence the microstructural characteristics, which in turn directly determine the mechanical properties of the forged components. Therefore, a thorough understanding of the microstructural evolution patterns and deformation mechanisms during hot deformation is essential for achieving precise control over microstructural morphology and mechanical properties. Figure 20 schematically illustrates the microstructural evolution associated with three typical dynamic softening mechanisms: DRV, CDRX, and DDRX. The underlying mechanisms and related research advances are discussed separately in the following sections.

### 4.1. Dynamic Recovery

DRV is a prevalent microstructural restoration mechanism during the high-temperature plastic deformation of Ti-55531 titanium alloy. At the initial stage of deformation, plastic straining induces rapid dislocation multiplication and entanglement, resulting in pronounced work hardening and a corresponding increase in flow stress. With increasing strain, thermally activated processes, including dislocation glide, cross-slip, and climb, facilitate dislocation rearrangement and partial annihilation, progressively leading to the formation of dislocation cells, LAGBs, and subgrain structures. As a dynamic balance is gradually established between dislocation generation and recovery, the microstructure may develop a relatively stable subgrain configuration, as schematically illustrated in Figure 20a. Therefore, DRV not only reduces the stored energy accumulated during deformation but also provides an essential microstructural basis for subsequent CDRX [79].

Warchomicka et al. [14] investigated the hot deformation behavior of Ti-55531 titanium alloy near the β-transus temperature and demonstrated that DRV of the β phase constitutes an important microstructural restoration mechanism in both the α + β two-phase and single-phase β regions. At relatively low strains, dislocation rearrangement and subgrain formation dominate the microstructural evolution. With further straining, the misorientation between adjacent subgrains progressively increases, potentially leading to the development of CDRX. Under the investigated conditions, DRV was identified as the primary restoration mechanism in the β phase, whereas CDRX developed progressively from the recovery-induced substructures with increasing accumulated strain.

The investigation by Wu et al. [65] on Ti-55531 titanium alloy in the single-phase β region similarly demonstrated that its microstructural restoration mechanisms are highly sensitive to deformation temperature and strain rate. Their hot compression experiments revealed that high temperatures and low strain rates favored the occurrence of DRX, whereas DRV remained important under other deformation conditions. These findings suggest that DRV and DRX are not mutually exclusive mechanisms. When the rate of dislocation recovery is sufficient to dissipate a substantial proportion of the stored energy, the microstructure may retain a predominantly subgrained configuration over an extended deformation interval. Conversely, when stored energy continues to accumulate and the conditions required for recrystallization are satisfied, CDRX or DDRX may be further activated. Zhang et al. [80] further investigated the influence of strain rate on the microstructural evolution of the β phase in Ti-55531 titanium alloy. They observed a higher density of residual dislocations within grains at relatively high strain rates, together with pronounced characteristics of DRV, indicating that deformation rate plays an important role in governing the balance between dislocation accumulation and microstructural restoration.

In addition to deformation parameters, alloy composition can influence DRV and its transition towards DRX. The β-stabilizing elements Mo, V, and Cr in Ti-55531 titanium alloy primarily affect the phase constitution and atomic diffusion conditions at a given deformation temperature by modifying β-phase stability and the T_β_. Furthermore, solute elements with relatively low diffusivities, particularly Mo, may exert a solute drag effect, thereby reducing grain boundary mobility and influencing subgrain coarsening and dislocation rearrangement [81]. Consequently, variations in nominal composition, elemental segregation, or initial heat treatment conditions may result in different degrees of DRV and DRX, even at comparable absolute deformation temperatures. Studies on Ti–Mo alloys have demonstrated that increasing the Mo content significantly increases the activation energy for β-grain growth, which has been attributed to the solute drag effect of Mo on grain boundary migration.

### 4.2. Continuous Dynamic Recrystallization

CDRX is essentially a progressive microstructural evolution process that develops from DRV. During deformation, dislocations initially rearrange to form subgrains and LAGBs. With continued straining, subgrains progressively absorb dislocations and undergo lattice rotation, resulting in an increase in the misorientation between adjacent subgrains. Consequently, some LAGBs gradually transform into HAGBs, eventually producing new recrystallized grains, as schematically illustrated in Figure 20b. CDRX generally exhibits no distinct boundary between nucleation and growth, but rather proceeds through the continuous evolution of deformation-induced substructures [82].

Warchomicka et al. [14] reported that DRV in the β phase of Ti-55531 titanium alloy could progressively evolve into CDRX at relatively high strains. Particularly at higher strain rates, the lattice orientations within β grains gradually changed towards the original grain boundaries, accompanied by the formation of new HAGBs near these boundaries, indicating that CDRX could proceed through progressive lattice rotation. Nevertheless, the authors also identified DRV as the predominant microstructural restoration mechanism throughout deformation. Accordingly, CDRX is more appropriately regarded as a further stage of microstructural evolution following the development of recovery-induced substructures, rather than a softening process entirely independent of DRV.

Fan et al. [52] further demonstrated that strain rate significantly influences the morphological characteristics of CDRX during hot deformation of coarse-grained Ti-55531 titanium alloy in the single-phase β region. With increasing strain rate, the intragranular substructures within the original β grains progressively transformed from approximately equiaxed morphologies into discontinuous band-like structures distributed near the original grain boundaries. At relatively high strain rates, both conventional CDRX and two-step CDRX were observed. In the latter mechanism, band-like substructures initially form and subsequently undergo fragmentation and grain refinement through lattice rotation and the development of HAGBs. Meanwhile, the formation of numerous deformation bands at high strain rates restricts the full development of the recrystallized microstructure. Therefore, an increase in strain rate does not necessarily lead to a higher overall degree of recrystallization. Although rapid dislocation accumulation can increase local stored energy and promote lattice rotation, the reduced deformation time may also constrain subgrain rearrangement and recrystallized grain growth. Zhang et al. [80] investigated the microstructural evolution of Ti-55531 titanium alloy at 900 °C under different strain rates. They found that both CDRX and DDRX could occur at low strain rates, whereas higher strain rates resulted in greater intragranular retention of dislocations, accompanied by characteristic features of DRV and CDRX. These observations further demonstrate that strain rate alters the relative contributions of different restoration and recrystallization mechanisms.

Meng et al. [83] investigated dynamic recrystallization during the forging of Ti-55531 titanium alloy across the β-transus temperature. Their results revealed pronounced CDRX characteristics in the β phase under conditions involving a relatively narrow temperature drop and a low cooling rate. During deformation, continuously accumulated dislocations undergo rearrangement through DRV, progressively forming subgrains and LAGBs. With further straining, the continued absorption of dislocations and progressive rotation of subgrains increase the boundary misorientation, promoting the transformation of some LAGBs into HAGBs and eventually producing fine recrystallized grains. The continuous evolution of intragranular orientation gradients and subgrain structures indicates that CDRX during deformation across different phase fields remains closely associated with the recovery-induced substructures, reflecting a progressive relationship between DRV and CDRX. Meanwhile, decreasing the final deformation temperature and increasing α-phase precipitation further affect β-grain boundary migration and substructural evolution. These findings suggest that CDRX under conditions involving deformation across the β-transus temperature is governed not only by temperature and strain rate but also by the cooling path and α-phase precipitation behavior.

**Figure 21:**
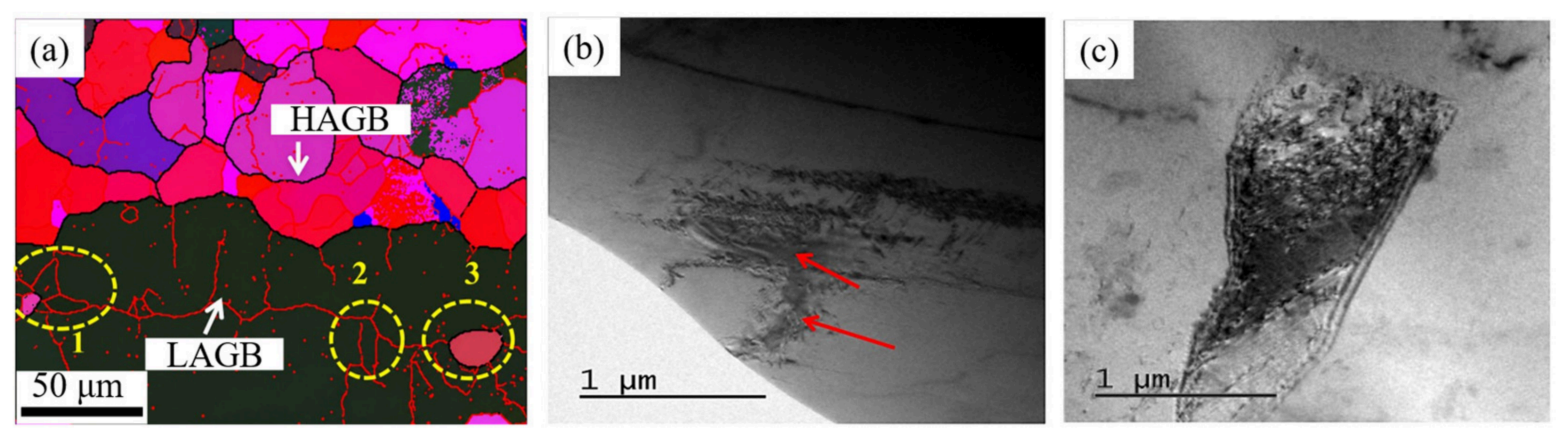
Microstructural characteristics associated with CDRX in Ti-55531 alloy deformed at 900 °C and a strain rate of 0.01 s^−1^: (**a**) EBSD Euler orientation map showing LAGBs (red lines) and HAGBs (black lines), where yellow dashed circles 1 and 2 highlight LAGBs formed through dislocation accumulation, and circle 3 indicates a region associated with the transformation of LAGBs into HAGBs; (**b**) TEM micrograph showing dislocation tangles and the development of dislocation walls and cells (red arrows); (**c**) TEM micrograph showing a newly formed recrystallized grain within an original β grain. Reproduced with permission from the authors of Ref. [61].

Cai [61] provided further microstructural evidence for the occurrence of CDRX in Ti-55531 alloy. At 900 °C and a strain rate of 0.01 s^−1^, numerous LAGBs were observed within the original β grains, with some subgrain boundaries exhibiting increased misorientation, suggesting the progressive transformation of LAGBs into HAGBs and the formation of fine recrystallized grains (Figure 21a). TEM observations further revealed dislocation tangles and the development of dislocation walls and cells within the β grains (Figure 21b), as well as newly formed recrystallized grains (Figure 21c). These observations are consistent with the characteristic evolution of CDRX, in which dislocation rearrangement promotes subgrain formation, followed by progressive lattice rotation and increasing boundary misorientation, ultimately facilitating the transformation of LAGBs into HAGBs.

In the α + β two-phase region, CDRX is additionally influenced by the presence of the second phase and deformation compatibility between the constituent phases. Xiang et al. [16] observed characteristic features of continuous orientation evolution in hot-compressed Ti-55531 titanium alloy. Their observations of intragranular substructures and continuous orientation changes indicate progressive subgrain rotation and the gradual transformation of LAGBs into HAGBs during deformation, which are consistent with the characteristic features of CDRX. Nevertheless, the presence of CDRX-related microstructural features does not necessarily indicate that CDRX is the dominant mechanism responsible for macroscopic flow softening.

### 4.3. Discontinuous Dynamic Recrystallization

DDRX is primarily characterized by localized bulging and migration of pre-existing HAGBs, accompanied by the nucleation and growth of new grains. Its driving force mainly arises from differences in stored energy or dislocation density across grain boundaries. During hot deformation, pronounced dislocation density gradients can develop near the original grain boundaries, enabling HAGBs to migrate through the absorption of surrounding dislocations and substructures. Meanwhile, LAGBs may form and subsequently evolve within the concave regions behind bulging grain boundaries, further facilitating the formation of recrystallized grains. Accordingly, grain boundary bulging, serrated grain boundaries, and fine recrystallized grains distributed along the original grain boundaries are generally regarded as characteristic microstructural features of DDRX, as schematically illustrated in Figure 20c [84].

**Figure 22:**
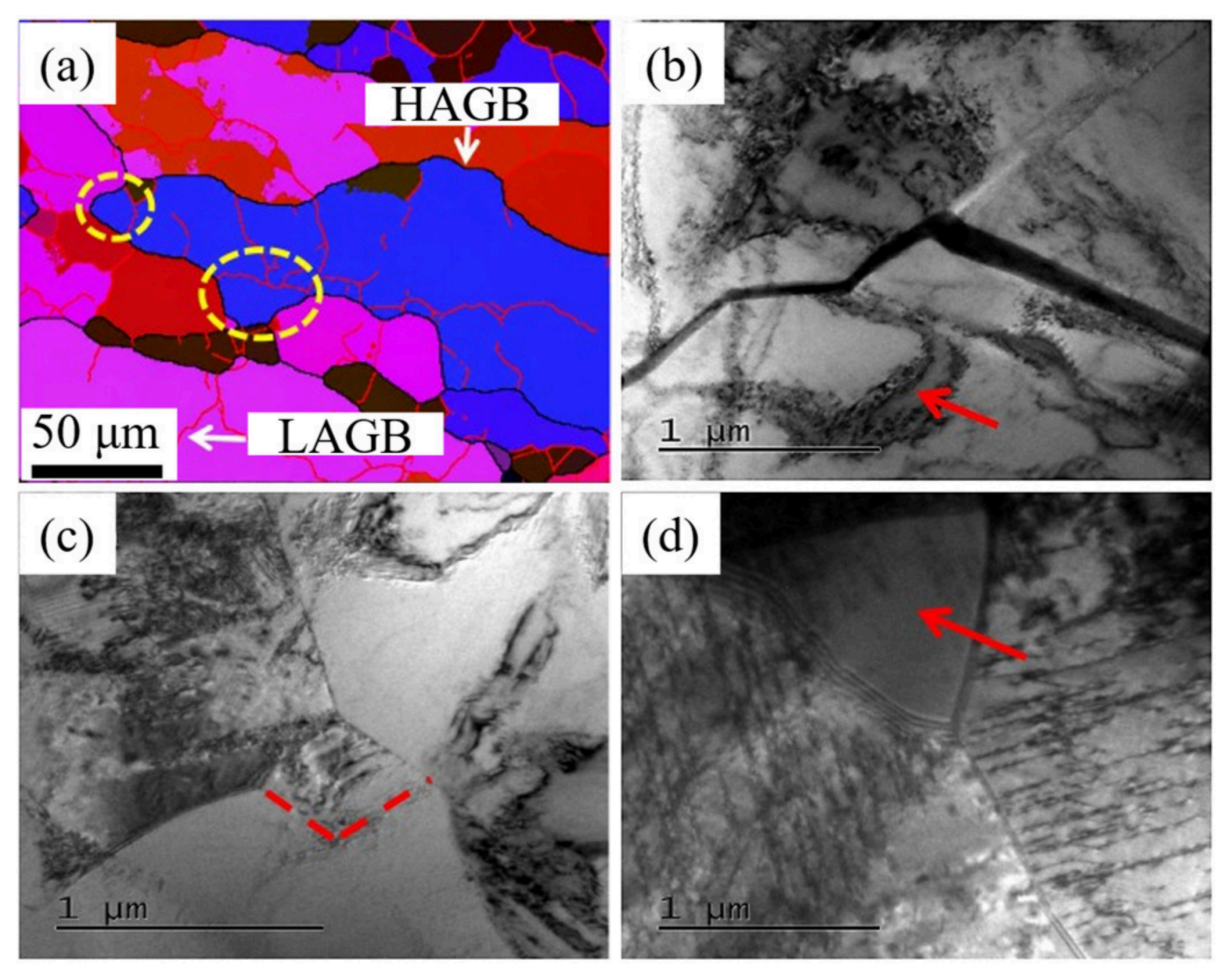
Microstructural characteristics associated with DDRX in Ti-55531 alloy at 900 °C and a strain rate of 0.01 s^−1^: (**a**) EBSD Euler orientation map showing LAGBs (red lines) and HAGBs (black lines), with yellow dashed circles highlighting localized grain boundary bulging and adjacent LAGBs; (**b**) TEM micrograph showing grain boundary bulging and dislocation wall formation (red arrow); (**c**) TEM micrograph showing a subgrain-like structure near a grain boundary triple junction (red dashed lines); (**d**) TEM micrograph showing a newly formed recrystallized grain near a grain boundary triple junction (red arrow). Reproduced with permission from the author of Ref. [61].

Xiang et al. [16] observed pronounced DDRX features during hot compression of Ti-55531 titanium alloy. Their results showed that localized bulging occurred along the original β-grain boundaries, accompanied by the formation of fine recrystallized grains in their vicinity. Misorientation analyses and the corresponding {111} pole figures revealed distinct crystallographic orientation differences between the newly formed grains and their parent grains, providing further evidence of grain boundary migration and new grain formation during DDRX. These observations indicate that DDRX preferentially occurs along the original β-grain boundaries and in adjacent regions exhibiting high strain gradients. Cai [61] further investigated the microstructural characteristics of DDRX in Ti55531 alloy. As shown in Figure 22a, localized bulging occurred along the original β-grain boundaries, accompanied by the formation of LAGBs on the opposite side of the bulged boundaries. TEM observations revealed dislocation accumulation and the formation of dislocation walls near the bulging grain boundaries (Figure 22b). With continued dislocation accumulation, these dislocation walls could progressively evolve into LAGBs, while subgrain-like structures were observed near grain boundary triple junctions (Figure 22c). Newly formed recrystallized grains were also identified at grain boundary triple junctions (Figure 22d). These observations provide further microstructural evidence supporting the occurrence of DDRX through localized grain boundary bulging and new grain formation. Fan et al. [52] investigated the influence of strain rate on DDRX behavior and reported that lower strain rates provided sufficient time for grain boundary migration, thereby facilitating the formation of DDRX grains. With increasing strain rate, the number of newly formed DDRX grains near deformation band boundaries and grain boundary triple junctions decreased, suggesting that higher strain rates restricted the full development of DDRX. Zhang et al. [80] further investigated the microstructural evolution of Ti-55531 titanium alloy at 900 °C under different strain rates. They found that CDRX and DDRX could occur concurrently at low strain rates, whereas higher strain rates resulted in greater retention of intragranular dislocations, accompanied by pronounced characteristics of DRV and CDRX. These findings further demonstrate that strain rate influences the relative contributions of different dynamic restoration and recrystallization mechanisms.

Meng et al. [83] investigated dynamic recrystallization during forging of Ti-55531 titanium alloy across the T_β_. Their results revealed that CDRX and DDRX could occur simultaneously in the β phase under conditions involving a relatively small temperature drop, whereas the extent of DDRX decreased as the final deformation temperature was further reduced. This behavior was attributed to reduced β-grain boundary mobility at lower temperatures and increased α-phase precipitation. The pinning effects exerted by fine α precipitates on dislocation motion and grain boundary migration further suppressed DRX. Therefore, DDRX during deformation across different phase fields is governed not only by temperature but also by the cooling path and α-phase precipitation behavior.

Consequently, the occurrence of DDRX requires both a sufficient stored-energy difference and adequate grain boundary mobility. Lower strain rates generally provide more time for grain boundary migration; however, excessively extensive DRV may continuously dissipate the locally accumulated stored energy. Conversely, although higher strain rates can rapidly increase dislocation density and local stored energy, the shortened deformation time may restrict grain boundary migration. Therefore, the dependence of DDRX on strain rate cannot be simply described as a monotonic increase or decrease. Instead, its evolution should be evaluated by considering the initial microstructure, the deformation temperature relative to the T_β_, and the phase constitution [85].

### 4.4. Competition and Transition Among DRV, CDRX, and DDRX

Existing studies indicate that DRV, CDRX, and DDRX in Ti-55531 titanium alloy do not occur within strictly separated processing regimes, but instead evolve and compete with one another as deformation proceeds. The initial stage of deformation is generally dominated by rapid dislocation multiplication and DRV, during which dislocation rearrangement leads to the formation of numerous LAGBs and subgrain structures. With increasing strain, continued dislocation accumulation may progressively increase the misorientation between adjacent subgrains, enabling CDRX through the gradual transformation of LAGBs into HAGBs. Meanwhile, if a sufficiently large stored-energy gradient develops near the original β-grain boundaries, and the deformation temperature and available time permit grain boundary migration, DDRX may occur through grain boundary bulging and migration. Accordingly, the relationship among these three mechanisms in Ti-55531 titanium alloy can be summarized as follows: DRV provides the substructural basis for subsequent microstructural evolution; CDRX proceeds primarily through progressive subgrain rotation and increasing boundary misorientation; and DDRX relies more strongly on the localized migration of pre-existing HAGBs [86].

Strain rate influences the competition between the rate of dislocation generation and the time available for microstructural restoration. At relatively high strain rates, rapid dislocation accumulation can generate high local stored energy and promote deformation band formation and lattice rotation. However, the reduced time available for microstructural rearrangement may restrict DRV, subgrain coarsening, and the full development of recrystallized grains. At lower strain rates, more time is available for dislocation climb, rearrangement, and grain boundary migration, thereby facilitating DRV while also providing favorable conditions for grain boundary migration-mediated DDRX. Consequently, reports of enhanced local CDRX features at higher strain rates and more extensive overall DRX at lower strain rates are not necessarily contradictory. Rather, these observations reflect differences in the relative kinetics of dislocation storage, recovery, and grain boundary migration. This competition is exemplified by Fan et al. [52], who observed enhanced CDRX-related features and deformation band formation at higher strain rates in a coarse-grained β microstructure, whereas Wu et al. [65] reported that high temperatures and low strain rates favored overall DRX. Similar competition among recrystallization mechanisms has also been reported in other near-β titanium alloys. Wang et al. [87] found that, during deformation of TC18 alloy in the α + β two-phase region, the recrystallization mechanism of the β phase progressively shifted from grain boundary migration-mediated DDRX towards intragranular substructure evolution-mediated CDRX with increasing strain rate, further demonstrating the distinct strain-rate sensitivities of different recrystallization mechanisms.

The influence of deformation temperature should be evaluated primarily in relation to the T_β_, rather than solely by comparing absolute temperatures. Differences in the predominant DRV, CDRX, and DDRX mechanisms reported across individual studies can be partly attributed to variations in deformation temperature relative to the respective T_β_. For near-β titanium alloys such as Ti-55531 titanium alloy, which contain relatively high concentrations of the β-stabilizing elements Mo, V, and Cr, even minor compositional variations may alter T_β_ and β-phase stability, thereby influencing the pathways of microstructural evolution during hot deformation [68]. To further compare the findings of different studies, Table 5 summarizes the predominant microstructural evolution mechanisms and the supporting evidence for their identification under different deformation conditions in Ti-55531 titanium alloy, highlighting the differences in dynamic softening mechanisms reported across these studies.

The presence of the α phase further complicates recrystallization behavior near the T_β_. On the one hand, deformation incompatibility and local stored-energy accumulation can develop near α/β interfaces, providing a driving force for substructure formation and localized recrystallization. On the other hand, the α phase can impede dislocation motion and β-grain boundary migration [88]. When substantial precipitation of fine α particles occurs during deformation across different phase fields, their pinning effect may restrict β-grain boundary migration, thereby suppressing DDRX and other recrystallization processes involving grain boundary motion. This effect was directly demonstrated by Meng et al. [84], while Xiang et al. [16] further showed that variations in the α-phase volume fraction themselves constitute an important source of flow softening in Ti-55531 titanium alloy during deformation in the α + β two-phase region. Therefore, the α phase should neither be regarded simply as a preferential nucleation site for recrystallization nor universally considered an inhibitor of recrystallization. Its actual influence depends on the volume fraction, size, morphology, and spatial distribution of the α phase relative to the β matrix [88].

Taken together, the above findings and the comparison presented in Table 5 indicate that the different conclusions regarding the predominant softening mechanisms in Ti-55531 titanium alloy, whether DRV, CDRX, or DDRX, are not necessarily contradictory. In addition to deformation temperature and strain rate, factors such as the initial β-grain size, initial α-phase fraction, deformation temperature relative to the T_β_, strain, cooling path, and the methods employed for microstructural characterization and mechanism identification can all influence the resulting interpretations. Flow stress curves or apparent activation energies alone are generally insufficient to distinguish DRV from different types of DRX. In contrast, EBSD characterization of subgrain structures, LAGB-to-HAGB evolution, lattice rotation, and bulging of original grain boundaries can provide more direct microstructural evidence. Therefore, comparisons among different studies should preferentially be conducted under comparable initial microstructural conditions, phase fields, and hot deformation parameters. Integrating flow behavior with microstructural evidence is essential to avoid directly equating mechanistic conclusions obtained under substantially different experimental conditions.

## 5. Application Fields

1. Aerospace sector

The Ti-55531 titanium alloy, owing to its excellent tensile strength, good hardenability, and favorable fracture toughness, is widely employed in primary load-bearing aircraft components. For instance, on the Airbus A380, this alloy is used for landing gear struts, engine pylon attachments, and wing-box connecting devices. It is also extensively utilized in core load-bearing structures of next-generation aircraft such as the Boeing 787 and Airbus A350 [89]. Furthermore, Ti-55531 titanium alloy is suitable for fuselage primary structures, flap track systems, and critical aero-engine components including compressor disks and blades that operate under demanding conditions [65].

2. Marine industry

In the marine industry, particularly in naval equipment and high-end civilian vessels, the Ti-55531 titanium alloy is regarded as a critical structural material owing to its exceptional seawater corrosion resistance and high specific-strength advantage. The alloy exhibits long-term durability in marine environments, effectively resisting pitting corrosion and stress corrosion cracking. Concurrently, its high strength and favorable fatigue resistance provide a solid foundation for equipment weight reduction and reliability enhancement. Its primary applications are in components with stringent safety and performance requirements, such as submarine pressure hulls, marine propulsion shafts, large propellers, as well as seawater piping systems, heat exchangers, and cooling systems [90].

3. High-end industrial applications

In the fields of high-end industrial and defense equipment, the application of the Ti-55531 titanium alloy continues to expand. Its high load-bearing capacity and impact resistance render it suitable for heavy machinery linkages, oil-drilling tools, and missile launchers. With the development of advanced forming technologies such as additive manufacturing, Ti-55531 titanium alloy has demonstrated substantial potential for the production of custom-designed components with complex geometries and demanding performance requirements, further broadening its application prospects [20].

In summary, the application scope of the Ti-55531 titanium alloy is continuously expanding with advancements in fabrication and processing technologies, spanning from large-scale civil aircraft structural components to deep-sea equipment, and has become an important material foundation supporting the lightweighting and high-performance development of high-end equipment.

## 6. Conclusions, Current Challenges and Future Perspectives

### 6.1. Conclusions

This review systematically summarizes research progress in the preparation processes, hot working behavior, and microstructural evolution of Ti-55531 titanium alloy and provides a comprehensive analysis of existing findings. The main conclusions are as follows:

(1) Different processing routes and heat treatment conditions significantly influence the microstructure and mechanical properties of Ti-55531 titanium alloy by controlling β-grain size, α-phase morphology and distribution, and phase constitution. The synergistic optimization of hot working parameters, initial microstructure, and subsequent heat treatment represents an important approach to achieving desirable microstructure–property combinations.

(2) Deformation temperature, strain rate, and strain collectively govern the flow behavior and microstructural evolution of Ti-55531 titanium alloy. Existing Arrhenius-type constitutive models, processing maps, and finite element simulations provide useful guidance for optimizing hot working parameters. However, their predictive capabilities are influenced by factors such as the initial microstructure, deformation phase field, and investigated experimental parameter ranges. Therefore, their predictions should be comprehensively evaluated in conjunction with experimental microstructural characterization.

(3) DRV, CDRX, and DDRX collectively contribute to microstructural evolution during hot deformation of Ti-55531 titanium alloy. DRV facilitates substructure formation through dislocation rearrangement and annihilation, providing the microstructural basis for subsequent CDRX. CDRX proceeds primarily through progressive subgrain rotation and the transformation of LAGBs into HAGBs, whereas DDRX is closely associated with localized bulging and migration of the original β-grain boundaries. These three mechanisms may coexist at different deformation stages and within localized regions, with their relative contributions jointly governed by deformation conditions, initial microstructure, β-phase stability, and α-phase precipitation behavior.

Overall, the microstructural evolution and mechanical properties of Ti-55531 titanium alloy are governed by the combined effects of processing conditions, phase evolution, and dynamic restoration and recrystallization mechanisms.

### 6.2. Key Issues and Challenges

In recent years, substantial research progress has been achieved in the preparation processes, hot-deformation behavior, and microstructural control of the Ti-55531 titanium alloy. However, several critical issues remain to be systematically addressed, with particular challenges persisting in the understanding of microstructural evolution mechanisms during hot deformation, multi-scale microstructural control, and the uniformity regulation of microstructures in complex components.

First, the competition and transitions among DRV, CDRX, and DDRX require further clarification. Existing studies indicate that the initial stage of hot deformation in Ti-55531 titanium alloy is generally accompanied by dislocation multiplication and DRV, during which dislocation rearrangement promotes the formation of subgrains and LAGBs. As deformation proceeds, progressive subgrain rotation facilitates the transformation of LAGBs into HAGBs, potentially leading to CDRX. Meanwhile, DDRX may occur when a sufficiently large stored-energy difference develops near the original β-grain boundaries and conditions are favorable for grain boundary migration. Overall, CDRX exhibits a clear evolutionary relationship with DRV, whereas DDRX is more sensitive to the migration of pre-existing HAGBs. These three mechanisms may therefore coexist at different deformation stages or within localized regions. Nevertheless, discrepancies remain among studies regarding their relative contributions and predominant mechanisms, which are closely associated with deformation temperature, strain rate, strain, temperature relative to the T_β_, and the initial material condition. Particularly during deformation across the T_β_, α-phase precipitation further influences dislocation motion, subgrain evolution, and β-grain boundary migration, thereby increasing the complexity of recrystallization behavior. At present, a systematic quantitative description of the critical conditions governing the transition from DRV to CDRX and the competition between CDRX and DDRX remains lacking, particularly with respect to the coupling between α-phase precipitation and β-phase recrystallization.

Second, significant difficulties persist in the multi-scale tailoring of complex microstructures. During thermomechanical processing and heat treatment, Ti-55531 titanium alloy typically develops a complex microstructural system consisting of the β matrix, primary α, secondary α, and substructures, with pronounced interactions among features at different scales. Most current studies remain focused on individual microstructural characteristics, and a comprehensive understanding of the synergistic evolution of multi-scale microstructures and their influence on microstructural stability is still inadequate. Third, the control of microstructural uniformity in large monolithic components remains a critical bottleneck limiting the engineering application of Ti-55531 titanium alloy. Large forgings exhibit pronounced temperature gradients, non-uniform strain distributions, and variations in cooling conditions, which tend to cause microstructural inhomogeneity across different regions, leading to fluctuations in mechanical properties and enhanced anisotropy. Most current studies are still based on laboratory-scale small specimens, and research on the internal microstructural evolution and uniformity control in large complex components remains scarce. Fourth, current investigations into the hot-working behavior of Ti-55531 titanium alloy still rely predominantly on experimental analysis. Although methods such as constitutive modeling, processing maps, and numerical simulation have found certain applications, their predictive capabilities for microstructural evolution remain limited. Establishing multi-scale coupled models relating thermomechanical parameters, microstructural evolution, and property responses to achieve precise modulation of microstructure and mechanical properties represents an important future research direction.

In summary, further deepening the understanding of microstructural evolution mechanisms during hot deformation, optimizing thermomechanical and heat-treatment processes, and strengthening multi-scale microstructural control and numerical simulation research are of great significance for promoting the engineering application of Ti-55531 titanium alloy in large critical load-bearing aerospace components.

### 6.3. Future Research Directions

In response to the above challenges, future research on the Ti-55531 titanium alloy can be pursued along the following directions.

(1) Quantitative Characterization and Unified Criteria for Hot Deformation Mechanisms

During hot deformation of Ti-55531 titanium alloy, DRV, CDRX, and DDRX generally coexist and compete, with their relative contributions governed by multiple factors, including deformation temperature, strain rate, strain, and deformation temperature relative to the T_β_. Future studies should integrate EBSD with TEM and in situ characterization techniques to systematically investigate dislocation evolution, subgrain formation and rotation, the transformation of LAGBs into HAGBs, and the migration of original β-grain boundaries. Such investigations would facilitate the establishment of quantitative criteria for distinguishing DRV, CDRX, and DDRX and identifying transitions among these mechanisms. Particular attention should be paid to the effects of variations in T_β_ and alloy composition on recrystallization behavior, as well as the coupling between α-phase precipitation and β-phase recrystallization during deformation across different phase fields. These efforts would help clarify the processing conditions under which individual softening mechanisms predominate and identify the critical conditions governing transitions between them.

(2) Multi-scale modeling and simulation

Multi-scale simulation techniques provide powerful tools for elucidating microstructural evolution mechanisms and predicting mechanical properties. Future work may integrate phase-field methods, cellular automata, and finite-element modeling to achieve coupled multi-scale simulations spanning dislocation evolution, grain growth, and macroscopic plastic deformation behavior. Establishing models that link thermomechanical parameters, microstructural evolution, and property prediction can provide a theoretical foundation for process design and optimization.

(3) Extended service property evaluations

As performance requirements for structural materials in aerospace applications continue to escalate, research on the service performance of Ti-55531 titanium alloy must be correspondingly expanded. Future investigations should place particular emphasis on the mechanical behavior and microstructural stability of the alloy at elevated temperatures, including high-temperature strength, creep resistance, and thermal stability. Concurrently, enhanced efforts are needed to study the performance evolution under complex service conditions, encompassing dynamic mechanical properties, impact loading responses, and fatigue–creep interactions, thereby providing a more robust theoretical and experimental basis for the long-term service of this alloy in critical aerospace load-bearing structures.

## Figures and Tables

**Table 1:** Tensile mechanical properties after β annealing and solution strengthening. Reproduced from Ref. [59] with permission of the author.

Heat Treatment Process	Direction	σ_b_/MPa	σ_0.2_/MPa	δ/%
β annealing + aging	Longitudinal	1170	1080	11.0
	Transverse	1170	1090	10.5
Solution + aging	Longitudinal	1360	1350	9.0
	Transverse	1380	1320	8.0

**Table 2:** Comparison of constitutive models developed for Ti-55531 titanium alloy under different hot deformation conditions.

References	Deformation Phase Field	Deformation Temperature (°C)	Strain Rate (s^−1^)	Constitutive Model	Prediction Accuracy
Li et al. [63]	α + β and single-phase β regions	700–1000	0.001–10	Arrhenius-type hyperbolic sine model	Relative error < 10%
Yang et al. [64]	α + β and single-phase β regions	650–800	0.01–1	Arrhenius-type hyperbolic sine model	Not reported
Wu et al. [65]	Single-phase β region	860–900	0.001–10	Arrhenius-type hyperbolic sine model	R > 0.98; AARE < 5.7%
Han [24]	α + β and single-phase β regions	730–900	0.001–10	Strain-compensated Arrhenius model	R = 0.989
Wu et al. [66]	α + β two-phase region	730–820	0.001–10	Strain-compensated Arrhenius model	R = 0.99; AARE = 7.94%
Zheng et al. [67]	α + β and single-phase β regions	730–900	0.001–10	Strain-compensated Arrhenius model	Mean relative error = 7.44%
Shokry et al. [68]	α + β two-phase region	760–840	0.001–1	Modified Fields–Backofen and Zerilli–Armstrong models	MFB: R = 0.996; AARE = 3.34%
Hu et al. [69]	α + β and single-phase β regions	750–900	0.01–10	Arrhenius-type and AHA-BP models	AHA-BP: R^2^ = 0.989; MAPE = 2.80%

**Table 3:** Comparison of processing maps of Ti-55531 titanium alloy under different hot deformation conditions.

Reference	Deformation Phase Field	Deformation Temperature (°C)	Strain Rate (s^−1^)	Favorable Processing Conditions	Flow Instability Characteristics
Han [24]	α + β and single-phase β regions	730–900	0.001–10	820–880 °C, 0.001–0.01 s^−1^	Flow instability occurs at low temperatures and high strain rates, with the instability region expanding as strain increases.
Wu et al. [65]	Single-phase β region	860–900	0.001–10	High temperatures and low strain rates favor DRX.	The severity of flow instability increases with increasing strain.
Dikovits et al. [71]	α + β and β	763–843	0.001–10	Evaluated using different instability criteria and microstructural observations	Flow localization associated with adiabatic heating; the flow-softening parameter α_i_ shows good agreement with microstructural observations.
Yang et al. [72]	α + β and single-phase β regions	790–940	0.01–1	Higher power dissipation efficiency is achieved at high temperatures and low strain rates.	No flow instability region
Zhu et al. [73]	α + β two-phase region	770–830	0.01–0.36	780–820 °C, 0.01–0.36 s^−1^	Varies with deformation conditions.
Hu et al. [69]	α + β and β	750–900	0.01–10	857–890 °C, 0.0125–0.0372 s^−1^ (ε = 0.8)	Instability mainly occurs at intermediate strain rates (0.1–1 s^−1^).

**Table 4:** Comparison of finite element modeling studies on hot deformation and microstructural evolution of Ti-55531 titanium alloy.

Reference	Simulation Object	Deformation Conditions	Constitutive and Microstructural Models	Finite Element Approach	Main Predicted Results
Lv et al. [77]	Cylindrical hot compression specimen	750–825 °C; 0.001–1 s^−1^; ε = 0.7	JMAK-type DRX kinetics model	DEFORM-3D; user-defined FORTRAN subroutine	DRX volume fraction and its spatial distribution
Wu et al. [65]	Large cylindrical workpiece subjected to non-isothermal forging	Φ100 mm × 150 mm; initial temperature of 890 °C; die temperature of 100 °C; 0.1 s^−1^; 60% height reduction	Physically based constitutive model coupled with models for the critical conditions, kinetics, and grain size evolution of DRX	DEFORM user subroutine; coupled thermo-mechanical–microstructural simulation	Forging load, temperature and strain distributions, DRX volume fraction, and grain size
Zheng et al. [67]	Cylindrical hot compression specimen	Model established over 730–900 °C and 0.001–10 s^−1^; FEM validation at 860 °C and 0.1 s^−1^	Arrhenius-type constitutive model coupled with models for DRX critical strain, volume fraction, and grain size	Finite element simulation	DRX volume fraction and grain size in different deformation regions
Zheng [75]	Ti-55531 titanium alloy torque arm forging	Two-stage forging process involving pre-forging and final forging	Constitutive model coupled with a DRX model	DEFORM-3D; forming process simulation and optimization	Material flow, forging load, forming defects, and processing parameters

**Table 5:** Comparison of dynamic restoration and recrystallization mechanisms reported for Ti-55531 titanium alloy and their supporting evidence.

Reference	Phase Field	Microstructural Evolution Mechanism	Main Supporting Evidence
Warchomicka et al. [14]	Near the β-transus temperature and in the single-phase β region	DRV predominates, accompanied by CDRX	Subgrain formation, progressive lattice rotation, and HAGB formation
Fan et al. [52]	Single-phase β region	CDRX predominates, accompanied by localized DDRX	Subgrain rotation, transformation of LAGBs into HAGBs, grain boundary bulging, and fine grains near grain boundaries
Wu et al. [65]	Single-phase β region	Competition between DRV and DRX	Flow behavior, activation energy, and recrystallization-related microstructural features
Zhang et al. [80]	Single-phase β region	Coexistence of CDRX and DDRX at low strain rates; DRV and CDRX features become more pronounced at higher strain rates	Intragranular dislocation retention, substructure evolution, and recrystallization-related grain boundary characteristics
Xiang et al. [16]	α + β two-phase region	Coexistence of CDRX and DDRX	Subgrain rotation, progressive misorientation accumulation, HAGB bulging, and fine grains near grain boundaries
Meng et al. [83]	Across the β-transus temperature	Coexistence of CDRX and DDRX, with reduced DDRX at lower temperatures	Continuous subgrain evolution, grain boundary migration, and the influence of α-phase precipitation

## Data Availability

No new data were created or analyzed in this study. Data sharing is not applicable to this article.
